# Wnt pathway activation by ADP-ribosylation

**DOI:** 10.1038/ncomms11430

**Published:** 2016-05-03

**Authors:** Eungi Yang, Ofelia Tacchelly-Benites, Zhenghan Wang, Michael P. Randall, Ai Tian, Hassina Benchabane, Sarah Freemantle, Claudio Pikielny, Nicholas S. Tolwinski, Ethan Lee, Yashi Ahmed

**Affiliations:** 1 Department of Genetics and the Norris Cotton Cancer Center, Geisel School of Medicine at Dartmouth College, Hanover, New Hampshire 03755, USA; 2 Department of Pharmacology and Toxicology, Geisel School of Medicine at Dartmouth College, Hanover, New Hampshire 03755, USA; 3 Department of Biological Sciences, Yale-NUS College, National University of Singapore, Singapore 138615, Singapore; 4 Department of Cell and Developmental Biology, Vanderbilt Ingram Cancer Center, and Vanderbilt Institute of Chemical Biology, Nashville, Tennessee 37232, USA

## Abstract

Wnt/β-catenin signalling directs fundamental processes during metazoan development and can be aberrantly activated in cancer. Wnt stimulation induces the recruitment of the scaffold protein Axin from an inhibitory destruction complex to a stimulatory signalosome. Here we analyse the early effects of Wnt on Axin and find that the ADP-ribose polymerase Tankyrase (Tnks)—known to target Axin for proteolysis—regulates Axin's rapid transition following Wnt stimulation. We demonstrate that the pool of ADP-ribosylated Axin, which is degraded under basal conditions, increases immediately following Wnt stimulation in both *Drosophila* and human cells. ADP-ribosylation of Axin enhances its interaction with the Wnt co-receptor LRP6, an essential step in signalosome assembly. We suggest that in addition to controlling Axin levels, Tnks-dependent ADP-ribosylation promotes the reprogramming of Axin following Wnt stimulation; and propose that Tnks inhibition blocks Wnt signalling not only by increasing destruction complex activity, but also by impeding signalosome assembly.

## Abstract (web summary)

Wnt/β-catenin signalling directs several developmental processes and is aberrantly activated in several cancers. Here the authors implicate Tankyrase—previously shown to target the scaffolding protein Axin for proteolysis—in early Wnt signalling by promoting the interaction between Axin and the Wnt co-receptor LRP6.

## Graphical abstract

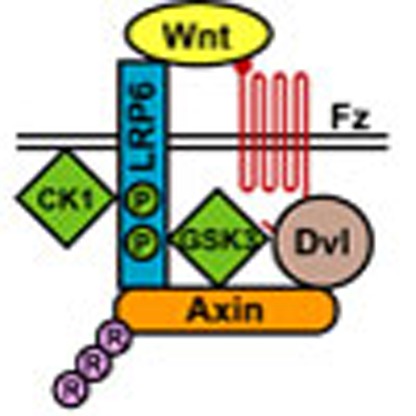

The Wnt/β-catenin signal transduction pathway directs fundamental processes during metazoan development and tissue homeostasis, whereas deregulation of Wnt signalling underlies numerous congenital disorders and carcinomas^1^,^2^. Two multimeric protein complexes with opposing functions—the cytoplasmic destruction complex and the plasma membrane-associated signalosome—control the stability of the transcriptional co-factor β-catenin to coordinate the state of Wnt pathway activation. In the absence of Wnt stimulation, β-catenin is targeted for proteasomal degradation by the destruction complex, which includes the two tumour suppressors: Axin and Adenomatous polyposis coli (APC), and two kinases: casein kinase 1α (CK1α) and glycogen synthase kinase 3 (GSK3)^3^,^4^,^5^,^6^. Engagement of Wnt with its transmembrane receptors, Frizzled and low-density lipoprotein receptor-related protein 5/6 (herein LRP6), induces rapid LRP6 phosphorylation, recruitment of Axin to phospho-LRP6, and assembly of the signalosome, which includes two other Axin-associated components, GSK3 and Dishevelled (Dvl)^7^,^8^,^9^,^10^,^11^,^12^,^13^,^14^. Signalosome assembly results in the inhibition of β-catenin proteolysis; consequently stabilized β-catenin promotes the transcriptional regulation of Wnt pathway target genes.

As a key component in both the destruction complex and the signalosome, Axin is tightly regulated. Under basal conditions, Axin is maintained at very low levels, and serves as the concentration-limiting scaffold for assembly of the destruction complex^15^,^16^. Following Wnt exposure, the rapid association of phospho-Axin with phospho-LRP6 (refs 7, 12, 14) triggers Axin dephosphorylation, inducing a conformational change that inhibits Axin's interaction with both the destruction and signalosome complexes^14^,^17^,^18^. Axin is subsequently degraded^7^,^18^,^19^,^20^,^21^,^22^,^23^,^24^; however, Axin proteolysis occurs several hours after Wnt exposure, and thus does not regulate Axin's essential role during the initial activation of the Wnt pathway.

The mechanisms that rapidly reprogram Axin from inhibitory to stimulatory roles following Wnt exposure remain uncertain. In current models, Wnt stimulation induces Axin's dissociation from the destruction complex, thereby promoting its interaction with the signalosome^1^,^5^,^6^,^13^,^14^,^18^,^22^,^25^,^26^,^27^. As Wnt stimulation induces Axin dephosphorylation^3^,^18^,^24^, decreased phosphorylation was postulated to facilitate the dissociation of Axin from the destruction complex^17^; however, recent work revealed that the interaction of Axin with LRP6 precedes Axin dephosphorylation, and that dephosphorylation serves to inhibit, rather than enhance this interaction^14^. Furthermore, some findings have challenged prevailing models, providing evidence that Axin's interaction with the destruction complex is not diminished upon Wnt stimulation^2^,^19^. Thus, whereas the rapid switch in Axin function following Wnt stimulation is essential for the activation of signalling, the underlying mechanisms remain uncertain.

During investigation of this critical process, we have discovered an unanticipated role for the ADP-ribose polymerase Tankyrase (Tnks) in the reprogramming of Axin activity following Wnt exposure. As Tnks-mediated ADP-ribosylation is known to target Axin for proteolysis^28^, small molecule Tnks inhibitors have become lead candidates for development in the therapeutic targeting of Wnt-driven cancers^29^,^30^,^31^. Here we identify a novel mechanism through which Tnks regulates Axin: by promoting Axin's central role in rapid Wnt pathway activation. We find that Wnt stimulation modulates Axin levels biphasically in both *Drosophila* and human cells. Unexpectedly, Axin is rapidly stabilized following Wnt stimulation, before its ultimate proteolysis hours later. In an evolutionarily conserved process, the ADP-ribosylated pool of Axin is preferentially increased immediately following Wnt exposure. ADP-ribosylation enhances Axin's association with phospho-LRP6, providing a mechanistic basis for the rapid switch in Axin function following Wnt stimulation. Our results thus indicate that Tnks inhibition not only increases basal Axin levels, but also impedes the Wnt-dependent interaction between Axin and LRP6, suggesting a basis for the potency of Tnks inhibitors in Wnt-driven cancers. Thus, Tnks not only targets Axin for proteolysis independently of Wnt stimulation, but also promotes Axin's central role in Wnt pathway activation, which may be relevant to the context-dependent activation of Wnt signalling and the treatment of Wnt-driven cancers with Tnks inhibitors.

## Results

### An *in vivo* assay for Axin regulation following Wg exposure

The precise onset of Wingless (Wg) /Wnt expression in fourteen segmental stripes during early *Drosophila* embryogenesis has provided a powerful *in vivo* model for the signalling events triggered by Wg (Supplementary Fig. 1)^32^,^33^. However, previous studies reported contradictory conclusions regarding Axin regulation, with the primary response to Wg exposure described as either Axin proteolysis^23^, or Axin recruitment from cytosol to plasma membrane^34^. Due to technical limitations in these studies, the analysis of Axin was restricted to several hours after the onset of Wg exposure. In addition, Axin was overexpressed at levels high enough to abrogate Wg signalling, which disrupts physiological regulation.

We reasoned that the conflicting conclusions in previous reports might have resulted from these experimental barriers. Therefore, we sought to develop a system that would permit an *in vivo* analysis of Axin at near-physiological levels immediately following Wg stimulation. Because endogenous levels of Axin in *Drosophila* embryos are too low to be visualized with Axin antibodies, we generated transgenes encoding Axin tagged with a V5 epitope (*Axin-V5)*, and used site-specific integration^35^,^36^ coupled with the UAS/Gal4 system^37^ to direct their expression at near-physiological levels. Expression of *Axin-V5* during early embryogenesis was induced with a maternal *α-tubulin* enhancer (*mat-Gal4*) that drives ubiquitous transcription^38^. In *Axin* null mutant embryos, Wg signalling is aberrantly activated throughout the ectoderm^39^, as revealed by the ectopic expression of both *engrailed* and *wg* (Supplementary Fig. 2a–h). Expression of *Axin-V5* in *Axin* null mutants completely prevented this constitutive activation of the Wg pathway, and critically, did not disrupt normal signalling (Supplementary Fig. 2i–n). Thus, Axin-V5 replaced the function of endogenous Axin, and was expressed at levels that were regulated physiologically. Further, in wild-type embryos expressing *Axin-V5*, the segmentally striped pattern of Wg, the resultant stabilization of Armadillo (Arm)/β-catenin in Wg-responding cells^40^, the Wg-dependent expression of *engrailed*^41^,^42^, and the specification of Wg-dependent cell fates, as revealed by the patterning of the larval cuticle^43^, were normal (Supplementary Fig. 3a–h).

To compare the relative levels of Axin-V5 and endogenous Axin, we examined embryonic lysates. Detection of endogenous Axin by immunoblotting of cell extracts had not been possible with existing Axin antibodies^18^. Therefore, we generated a new Axin antibody that allowed sensitive detection of endogenous Axin, and confirmed its specificity using RNAi-mediated *Axin* knockdown (Supplementary Fig. 3i). We used this Axin antibody to immunoblot lysates from wild-type embryos expressing *Axin-V5* (Supplementary Fig. 3j). Axin-V5 was distinguished from endogenous Axin by its slower migration rate in electrophoresis, resulting from the V5 tag. Immunoblotting revealed that Axin-V5 was not overexpressed relative to the level of endogenous Axin; instead, Axin-V5 levels were lower than the level of endogenous Axin both before and after the onset of Wg expression (Supplementary Fig. 3j–l). We conclude that Axin levels were increased by less than twofold following the expression of Axin-V5.

### Biphasic regulation of Axin following Wg and Wnt exposure

**Figure 1:**
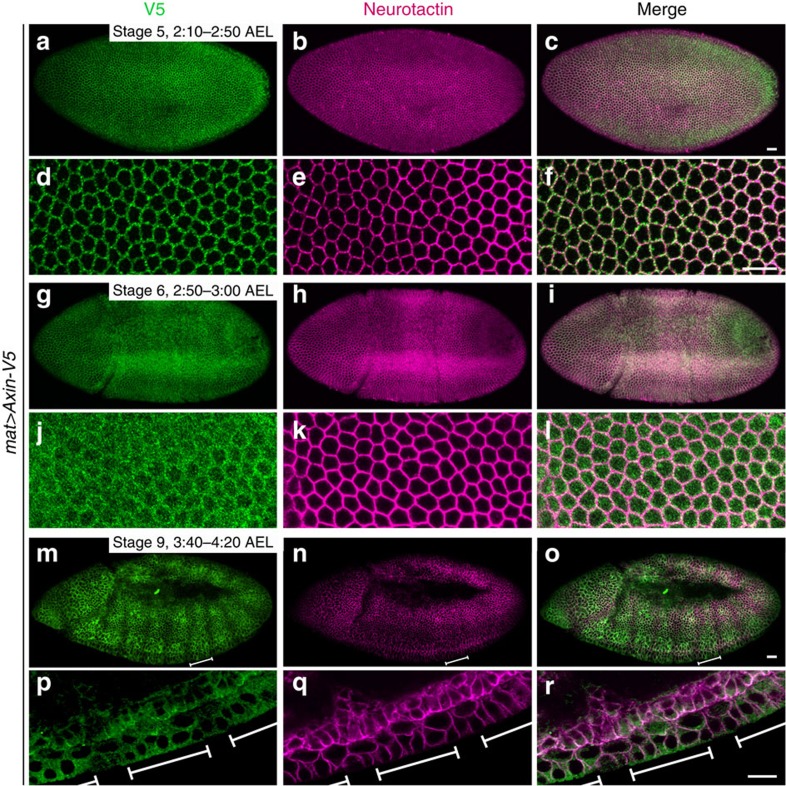
Rapid appearance of Axin in segmental stripes following Wg exposure. (**a**–**l**) Axin is distributed uniformly in the embryonic ectoderm before and at the onset of Wingless (Wg) expression. Confocal images of embryos expressing *Axin-V5* driven by the *mat-Gal4* driver. Genotype left, antibodies top. Embryonic stage and developmental time in hours after egg lay (AEL) are indicated at the top right of **a**,**g** and **m**. Anterior left, dorsal up. In stage 5 and 6 embryos, Axin-V5 and the transmembrane protein Neurotactin are distributed uniformly throughout the ectoderm (**a**–**l**). Higher magnification images (**d**–**f** and **j**–**l**). (**m**–**r**) Axin is distributed in segmental stripes after the onset of Wg expression. By stage 9, Axin-V5 accumulates in segmental stripes with increased staining intensity (**m**–**o**). Higher magnification images (**p**–**r**). White bars indicate the width of a single Axin stripe. Scale bar, 25 μm.

**Figure 2:**
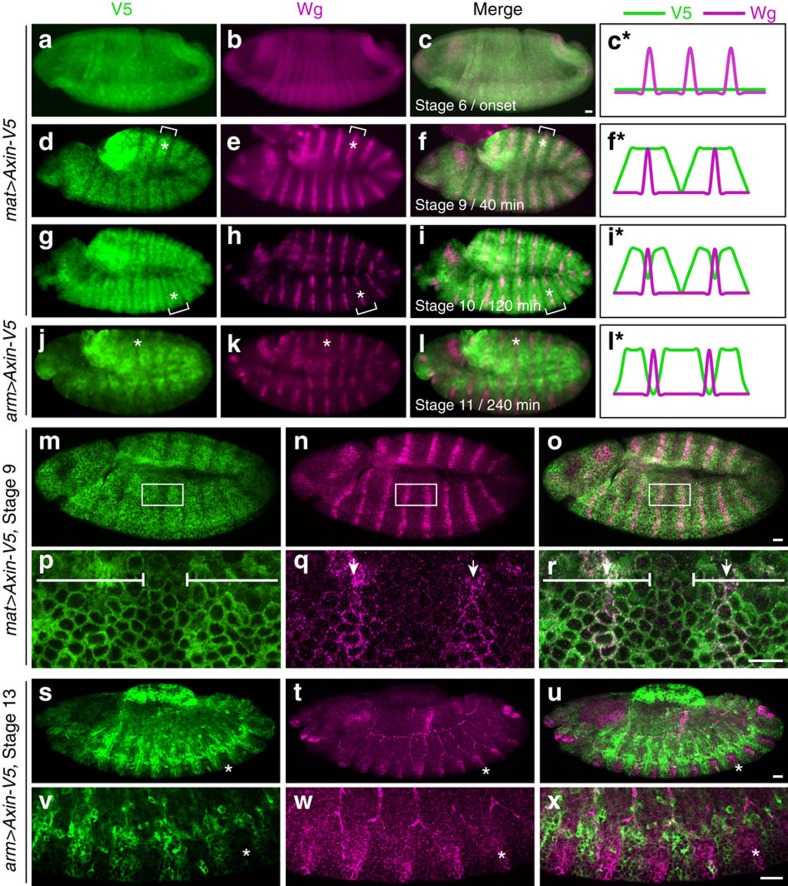
Axin is rapidly stabilized and subsequently destabilized following Wg exposure. (**a**–**c**) Axin is distributed uniformly in the ectoderm at the onset of Wg exposure. Embryos expressing *Axin-V5* driven by *mat-Gal4 (mat>Axin-V5)* stained with V5 and Wg antibodies. Stage of embryonic development and corresponding time relative to the onset of Wg expression is indicated at bottom left of **c**,**f**,**i**,**l**. At 3 h of development (stage 6), Axin-V5 is distributed uniformly throughout the embryonic ectoderm and the initial expression of Wg in segmental stripes is weak. (**d**–**f**) Axin levels increase rapidly following Wg exposure. By 30 to 40 min after the onset of Wg expression (late stage 8 to stage 9), Axin-V5 is distributed in wide segmental stripes (indicated by brackets) that overlap the narrow Wg stripes (asterisks). (**g**–**l**) Axin levels subsequently decrease, beginning at 2 h after Wg exposure. (**g**–**i**) Approximately 120 min after onset of Wg expression, mid-stage 10 embryos. During this transitional stage, the Axin-V5 level is decreased precisely at the position of the Wg stripes (asterisks), but remains elevated in the neighbouring cells (brackets). (**j**–**l**) Embryos expressing *Axin-V5* driven by *arm-Gal4 (arm>Axin-V5)* stained with V5 and Wg antibodies. *arm-Gal4* drives ubiquitous transcription at late embryonic stages, and thus allows analysis of Axin regulation in stage 11 embryos. By 240 min after the onset of Wg exposure, the Axin-V5 level is markedly decreased at the position of the Wg stripes. Wg (asterisks) and Axin stripes are spatially juxtaposed. (c*–l*) Schematic illustration of the spatial relationship between Axin-V5 and Wg with respect to the onset of Wg exposure. (**m**–**r**) Axin levels increase rapidly in response to Wg exposure. Confocal images of stage 9 embryo (**m**–**o**) and higher magnification images of boxed region (**p**–**r**). Axin stripes (white bars) are centered directly over Wg expressing cells (arrows). (**s**–**x**) Axin levels are decreased by several hours after Wg exposure. Confocal images of stage 13 embryo expressing *Axin-V5* with the *arm-Gal4* driver. By this time, Axin and Wg stripes are spatially juxtaposed (**s**–**u**), as shown in higher magnification images (**v**–**x**). Asterisks indicate the position of Wg stripe. For all images, anterior left, dorsal up. Scale bar, 25 μm.

Thus, expression of these *Axin* transgenes in wild-type embryos enabled an *in vivo* analysis of Axin at near-physiological levels before, during, and after the onset of Wg stimulation. We found that during the first 3 h of embryogenesis, before and during the onset of Wg expression, Axin was distributed uniformly throughout the ectoderm (Figs 1a–l and 2a–c). However, within 30 min after the onset of Wg expression in segmental stripes, a major change in Axin was observed, starting in late stage 8 and persisting throughout stage 9. Axin was no longer distributed uniformly, but instead was present in wide segmental stripes of increased intensity (Fig. 1m–r; Supplementary Fig. 4). The Axin fluorescence intensity in these stripes was two- to threefold higher than that in the interstripes (Supplementary Fig. 4). Moreover, the temporal appearance of the Axin stripes was coincident with the stabilization of Arm/β-catenin in segmental stripes^40^ (Supplementary Figs 5d–f and 6a–c), suggesting that the Axin stripes were an early indicator of the initial cellular response to Wg stimulation.

To determine the relative position of the segmental stripes of Axin and Wg, we co-stained embryos expressing *Axin-V5* with V5 and Wg antibodies. Within 30 min after the onset of Wg expression in segmental stripes, all Axin stripes were centered over Wg stripes (Fig. 2d–f,m–r; Supplementary Fig. 5a–c). As Wg is not required for embryonic segmentation^43^, we tested whether Wg was important for generating the segmental stripes of Axin. In contrast with wild-type embryos, no Axin stripes were observed in *wg* null mutants; instead, the Axin-V5 staining was uniform (Supplementary Fig. 6d–g). Thus, Wg is required for the differential regulation of Axin in the embryonic ectoderm.

To rule out the possibility that the segmental Axin stripes were caused artifactually by the V5 epitope tag, we generated transgenic flies expressing GFP-tagged Axin; *Axin-GFP* was also expressed using the *mat-Gal4* driver. Before, during, and after the onset of Wg expression, the Axin-GFP and Axin-V5 patterns were identical, as were the kinetics of Wg-dependent changes in their distribution (Supplementary Fig. 7). These data supported the hypothesis that within 30 min after Wg exposure, Axin is tightly regulated in Wg-responding cells, and that the rapid appearance of Axin in stripes of increased intensity coincides with the initial activation of the pathway.

To further test this hypothesis, we examined endogenous Axin levels in lysates from wild-type embryos. Embryos were collected at developmental stages that just preceded (1.5 to 2.5 h of development) or followed (3.5 to 4.5 h of development) the onset of Wg expression. To monitor the initial activation of the Wg pathway, we examined the accumulation of phosphorylated Arrow/LRP6 with an antibody directed against phosphothreonine^1572^ in human LRP6 (ref. 44), a residue that is conserved in *Drosophila* (Supplementary Fig. 8a). RNAi-mediated *arrow* knockdown in S2R+ cells confirmed the specificity of this signal (Supplementary Fig. 8b,c). After the onset of Wg stimulation, phospho-Arrow levels increased and remained elevated (Supplementary Fig. 9a).

We found that Axin levels increased by approximately threefold after Wg stimulation (Supplementary Fig. 9a,b; stage 9). The increase in Axin levels likely resulted from increased protein stability, as the *Axin* gene is not a transcriptional target of the Wg pathway, and instead *Axin* transcripts are expressed ubiquitously^39^. These findings provided further evidence that Axin is unexpectedly stabilized following Wg stimulation, and supported the kinetics of Axin stabilization observed in the embryos by immunostaining.

In addition, we found that after the initial increase in Axin during the early response to Wg exposure, Axin levels subsequently decreased (Supplementary Fig. 9a,b; stage 13, 9h 20 min to 10h 20 min of development), as reported previously for mammalian Axin^18^,^19^,^22^,^23^,^24^. To investigate the kinetics of this process, we examined the *in vivo* pattern of embryonic Axin stripes. We found that the Axin stripes persisted for 80 to 100 min following Wg exposure (Fig. 2d–f,m–r; Supplementary Figs 5a–c and 7m–o; late stage 8 to stage 9). After that time, Axin levels decreased, initially in cells exposed to the highest levels of Wg (Fig. 2g–i, 2 h following Wg exposure; mid-stage 10), and subsequently, in their neighbours (Fig. 2j–l,s–x, 4 h following Wg exposure; Stage 11-13). Thus, when Axin was stabilized in the early phase following Wg exposure, Axin and Wg stripes overlapped (Fig. 2d–f,m–r; Supplementary Figs 5a–c and 7m–o). By contrast, when Axin was destabilized in the delayed phase, Axin and Wg stripes were spatially juxtaposed (Fig. 2j–l,s–x), as observed previously^23^. In summary, our unprecedented ability to visualize Axin before, during, and after the onset of Wg exposure revealed that Axin is regulated biphasically in response to Wg stimulation *in vivo*, which reconciles the contradictory conclusions in previous reports^23^,^34^.

To determine whether the biphasic regulation of Axin in response to Wg stimulation is evolutionarily conserved, we examined Axin1 levels in cultured human cells exposed to Wnt (Supplementary Fig. 9c–f). Treatment of two cell lines, HEK293T or SW480, with Wnt3A protein resulted in rapid pathway activation, with the accumulation of phospho-LRP6 within 15 to 30 min (Supplementary Fig. 9c,e). In both cell lines, the levels of endogenous Axin1 protein increased within 15 to 30 min following Wnt3A treatment (Supplementary Fig. 9c–f). This increase in Axin1 likely resulted from increased protein stability, as the *Axin1* gene is not a transcriptional target of the Wnt pathway^45^,^46^, and the increased Axin1 protein level was observed within 15 min of Wnt exposure. By 2 h after Wnt treatment, Axin1 levels decreased (Supplementary Fig. 9c–f), as reported previously^18^,^24^. These findings suggested that the initial stabilization and subsequent destabilization of Axin in response to Wnt exposure, and the kinetics of this response, are evolutionarily conserved.

### Tnks promotes Axin stabilization following Wg exposure

As Tnks is known to target Axin for proteolysis^28^, we sought to determine whether Tnks regulates Axin before and/or following Wg exposure. The Tnks-dependent proteolysis of Axin requires direct interaction of Tnks with a small region in the Axin amino terminus, the Tnks-binding domain (TBD; refs 28, 47, 48). To determine whether the evolutionarily conserved TBD facilitates the degradation of *Drosophila* Axin, we generated an *Axin-V5* transgene with a 21 amino-acid deletion that eliminates the TBD (AxinΔTBD; Supplementary Fig. 10a,b). To enable their direct comparison, the *Axin-V5* and *AxinΔTBD-V5* transgenes were integrated at the same genomic site (*attP33)*, and were expressed using the same *mat-Gal4* driver. By comparison with Axin-V5, the levels of AxinΔTBD were increased by three- to fourfold (Supplementary Fig. 10c–g), even before the onset of Wg expression (0–2 h of embryogenesis, up to stage 4; Supplementary Fig. 10c–e). We conclude that the TBD is important to control the basal levels of *Drosophila* Axin independently of Wg exposure.

**Figure 3:**
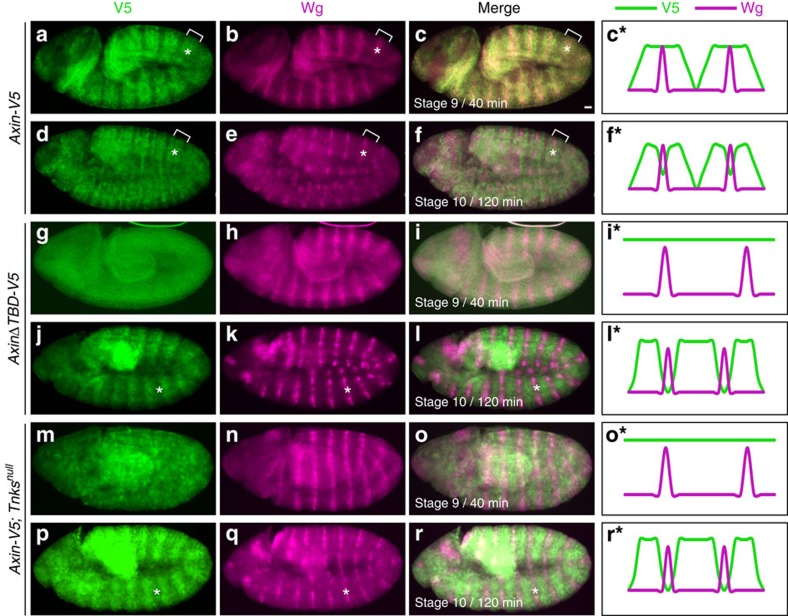
Tnks promotes Axin regulation during the early phase, but is dispensable for Axin proteolysis during the delayed phase after Wg exposure. (**a**-**f**) Wild-type Axin is initially stabilized and subsequently degraded following Wg exposure. Stage 9 and mid-stage 10 embryos expressing *Axin-V5* driven by the *mat-Gal4* driver, co-stained with V5 and Wg antibodies. Genotypes left, antibodies top. (**a**–**c**) By 40 min after the onset of Wg exposure (stage 9), Axin-V5 is distributed in wide segmental stripes (brackets) that overlap narrow Wg stripes (asterisks). (**d**–**f**) By 120 min after the onset of Wg exposure (mid-stage 10), Axin-V5 staining is decreased in cells expressing Wg (asterisks). (**g**–**l**) The Tnks-binding domain in Axin is required for the initial stabilization of Axin induced by Wg exposure, but dispensable for the subsequent Wg-dependent Axin proteolysis. (**g**–**i**) In stage 9 embryos, AxinΔTBD-V5 staining is uniformly high throughout the embryonic ectoderm; no segmental stripes are present. (**j**–**l**) In mid-stage 10 embryos, the AxinΔTBD-V5 levels are decreased in cells expressing Wg (asterisks). Axin stripes are spatially juxtaposed with Wg stripes. (**m**–**r**) Tnks is required for the initial stabilization of Axin induced by Wg exposure, but dispensable for the subsequent Wg-dependent Axin proteolysis. (**m**–**o**) In stage 9 *Tnks* null mutant embryos, Axin-V5 staining is uniformly high in all ectodermal cells. (**p**–**r**) In mid-stage 10 *Tnks* null mutant embryos, Axin-V5 is decreased in cells expressing Wg (asterisks). Axin stripes are spatially juxtaposed with Wg stripes. Scale bar, 25 μm. Schematic illustration of spatial relationship of Axin-V5 and Wg with respect to the onset of Wg expression in c*–r*.

To determine whether the TBD has additional roles in Axin regulation following Wg exposure, we analysed embryos expressing *AxinΔTBD* after the onset of Wg expression. Unexpectedly, and in contrast with Axin-V5 (Fig. 3a–c), AxinΔTBD-V5 was not present in segmental stripes 30 min after Wg exposure; instead, AxinΔTBD levels were increased uniformly in all ectodermal cells (Fig. 3g–i; Supplementary Fig. 11a–c; late stage 8 and stage 9). Thus, the early stripes of Axin that form in response to Wg exposure were dependent on the TBD; deletion of the TBD resulted in the aberrant Axin stabilization in all cells.

To determine whether the TBD is also required for the subsequent degradation of Axin induced by Wg, we examined embryos at later developmental stages (Fig. 3j–l). By comparison with their neighbours, the levels of AxinΔTBD were decreased in Wg-responding cells 2 h after Wg exposure, with kinetics similar to those observed for wild-type Axin (Fig. 3d–f,j–l; Supplementary Fig. 12i–k; mid-stage 10). These findings indicated that the TBD is not required for the Wg-dependent proteolysis of Axin. Therefore, we hypothesized that Tnks facilitates the stabilization of Axin following Wg exposure, but is dispensable for the subsequent Axin degradation.

To test this hypothesis, we examined *Tnks* null mutant embryos expressing *Axin-V5*. In contrast with wild-type embryos, no early stripes of Axin were present in *Tnks* mutants; instead, Axin levels were uniformly high throughout the embryonic ectoderm (Fig. 3m–o; Supplementary Fig. 11d–f). Two hours after Wg exposure, the levels of Axin decreased in Wg-responding cells in *Tnks* mutants, as we had observed in wild-type embryos (Fig. 3d–f,p–r). Thus, the same phenotype was present in embryos expressing *AxinΔTBD* or in *Tnks* null mutant embryos: the early Axin stripes were absent, whereas the late Axin stripes were present. These data indicated that in the absence of Tnks, the initial Wg-dependent regulation of Axin did not occur, and Axin was aberrantly stabilized in all cells, but that the subsequent Wg-dependent Axin proteolysis occurred with normal kinetics.

**Figure 4:**
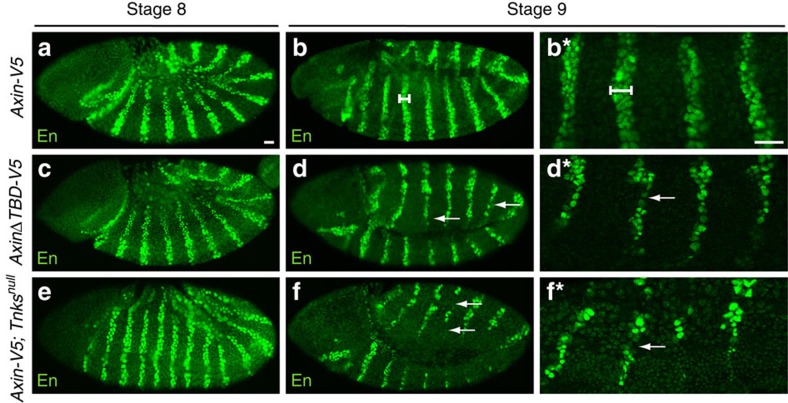
Tnks promotes the activation of Wg signalling. Tnks and the TBD in Axin promote Wg signalling. Confocal images of stage 8 or 9 embryos stained with Engrailed (En) antibody. Genotypes left, developmental stage indicated on top. (**a**,**b**) In wild-type embryos expressing *Axin-V5*, En is present in stripes that are 2 to 3 cells in width (b*, white bar), at both 10 min after the onset of Wg expression (stage 8) and 40 min after the onset of Wg (stage 9). (**c**,**d**) In embryos expressing *AxinΔTBD-V5*, the initiation of En stripes (stage 8) is normal (**c**), but En stripes decay in 68% (*n*=50) of embryos (**d**), as revealed by the aberrantly narrowed width by stage 9 (d*, arrow). (**e**,**f**) In *Tnks* null mutant embryos expressing *Axin-V5*, En expression is normal at stage 8 (**e**), but decays by stage 9 in 75% (*n*=36) of embryos (f*, arrow). Images in b*,d* and f* are higher magnification views of embryos in **b**,**d**, and **f** respectively. In all images, anterior left, dorsal up. Scale bar, 25 μm.

To determine whether the regulation of Axin at the earliest times after Wg exposure promotes activation of the Wg pathway, we analysed the expression of the transcription factor Engrailed. During early embryogenesis, the initial transcription of *engrailed* is not dependent on Wg; however, Wg signalling is required subsequently to maintain *engrailed* expression^41^,^42^. The expression of *engrailed* is among the earliest readouts for the activation of Wg signalling. Upon expression of *Axin-V5* in either wild-type or *Axin* null mutant embryos, both the onset and maintenance of *engrailed* expression in segmental stripes were normal (Fig. 4a,b and Supplementary Fig. 2m; compare with Supplementary Fig. 2b). In contrast, upon expression of *AxinΔTBD-V5* in either wild-type or *Axin* null mutant embryos, the initiation of *engrailed* expression was normal, but the maintenance of *engrailed* expression was markedly diminished, as revealed by the decreased width of Engrailed stripes (Fig. 4c,d; Supplementary Fig. 12m), indicating defects in Wg signalling. Similarly, examination of larval cuticles following expression of *Axin-GFP* revealed normal epidermal patterning, in which regions of naked cuticle alternated between ventral segmental denticle belts (Supplementary Fig. 13a). In contrast, a segment polarity phenotype was observed upon expression of *AxinΔTBD-GFP* (Supplementary Fig. 13b), in which the ventral epidermis formed denticles in place of naked cuticle^43^. These findings suggested that Tnks promotes not only the initial Wg-dependent regulation of Axin, but also the activation of Wg signalling.

We tested this hypothesis by examining Wg pathway activation in *Tnks* null mutant embryos expressing *Axin-V5*. In *Tnks* mutant embryos expressing *Axin-V5*, the initiation of *engrailed* expression was normal (Fig. 4e; stage 8), but its maintenance was markedly disrupted (Fig. 4f; stage 9). Therefore, the same defects in Wg pathway activation were observed in *Tnks* mutant embryos expressing *Axin-V5* and in wild-type embryos expressing *AxinΔTBD-V5*. Nonetheless, no defects in *engrailed* expression were observed in *Tnks* mutants (Supplementary Fig. 14), and these mutants were viable under standard laboratory conditions^49^,^50^,^51^. These findings revealed that a less than twofold increase in Axin levels by the expression of *Axin-V5* (Supplementary Fig. 3l) uncovers the importance of Tnks in promoting Wg signalling during embryogenesis, and suggested that the regulation of Axin by Tnks following Wg exposure can be functionally compensated until Axin reaches a critical threshold level. Altogether, these findings indicated that Tnks promotes not only the stabilization of Axin after Wg exposure, but also the activation of Wg signalling.

### Tnks promotes Wg signalling through a novel mechanism

**Figure 5:**
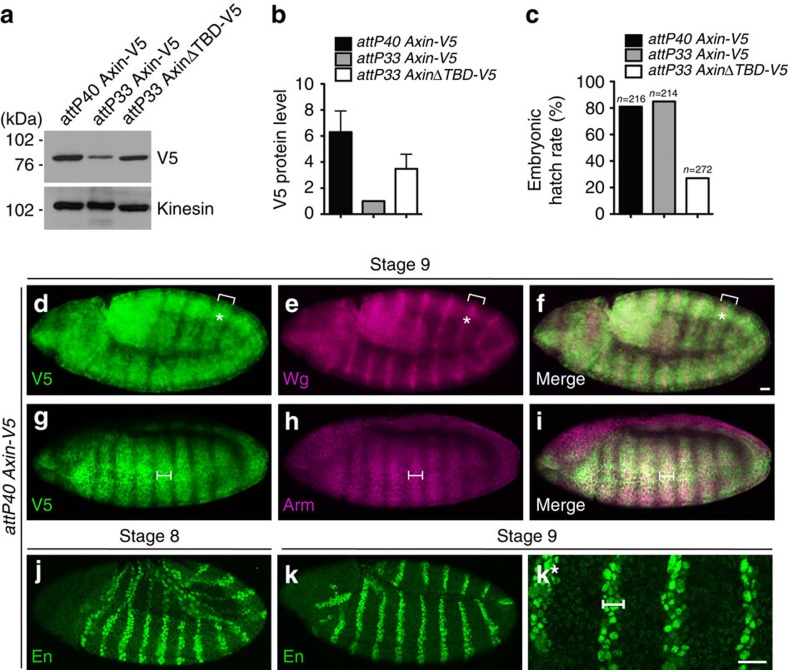
In addition to its known role in Axin proteolysis, Tnks promotes Wg pathway activation through a novel mechanism of Axin regulation. (**a**-**b**) Generation of transgenic flies with wild-type Axin at levels higher than those resulting from loss of Tnks-dependent Axin proteolysis. (**a**) Immunoblot analysis of lysates from embryos expressing either *Axin-V5* or *AxinΔTBD-V5* integrated at the *attP33* or the *attP40* site. Lysates were prepared from embryos collected at 0–2 h of development, before the onset of Wg expression. Levels of V5 from *attP33 AxinΔTBD-V5* are higher than *attP33 Axin-V5* when expressed under the same conditions. Integration of the wild-type *Axin-V5* transgene at a different genomic site, *attP40*, results in levels that are higher than *attP33 AxinΔTBD-V5*. Kinesin was used as a loading control. (**b**) Quantification of relative levels of indicated protein from experiments shown in **a**. Error bars represent s.e.m. of three independent experiments. *P*=0.0348 (one-way ANOVA with Kruskal–Wallis test). (**c**) The hatch rate of embryos expressing *attP40 Axin-V5*, *attP33 Axin-V5* or *attP33 AxinΔTBD-V5* with the *mat-Gal4* driver. By comparison with embryos expressing *attP33 Axin-V5* or *attP40 Axin-V5*, the hatch rate is reduced by expression of *attp33 AxinΔTBD-V5*. At least 200 embryos of each genotype were analysed, as indicated. (**d**–**i**) An increase in wild-type Axin, above the levels resulting from loss of Tnks-dependent Axin proteolysis, is compatible with the rapid stabilization of Axin following Wg exposure. (**d**–**f**) In stage 9 embryos expressing *attP40 Axin-V5* driven by *mat-Gal4*, Axin-V5 accumulates in segmental stripes (brackets) that overlap the Wg stripes (asterisks). (**g**–**i**) Co-staining with V5 and Arm antibodies shows that Axin-V5 accumulates normally in Wg-responding cells, as revealed by co-localization with cells that accumulate Arm (white bars). (**j**,**k**) An increase in wild-type Axin, above the levels resulting from loss of Tnks-dependent Axin proteolysis, is compatible with the activation of Wg signalling. En expression is normal at both stage 8 (**j**) and stage 9 (**k**). A higher magnification view of the En stripes in **k** is shown (k*, white bar indicates the width of a single En stripe). Scale bar, 25 μm.

We sought to elucidate the mechanism through which Tnks regulates Axin and the activation of Wg signalling. In the current model, Tnks promotes Axin degradation, limiting the activity of the destruction complex, and thereby facilitating the activation of Wg signalling^28^. Alternatively, Tnks might also regulate the rapid transition of Axin following Wg stimulation. To distinguish between these two possibilities, we tested whether an increase in the basal levels of Axin, comparable to the higher levels present in AxinΔTBD or in *Tnks* mutants, was sufficient to inhibit Axin regulation or Wg pathway activation. We generated transgenic flies in which the *Axin-V5* transgene was integrated at a different site in the genome (*attP40*), which is known to result in higher expression levels than the original site (*attP33*)^35^. Indeed, immunoblotting of lysates revealed that the basal levels of Axin in embryos expressing *Axin-V5* integrated at the *attP40* site were significantly higher than the Axin levels in embryos expressing either *Axin-V5* or *AxinΔTBD* integrated at the *attP33* site (Fig. 5a,b). Similarly, comparison of fluorescence intensity revealed that levels of *attP40 Axin-V5* were higher than *attP33 Axin-V5, attP33 AxinΔTBD-V5*, or *Tnks* null mutant embryos expressing *attP33 Axin-V5* (Supplementary Fig. 15).

However, despite the increased levels of Axin present in embryos expressing *attP40 Axin-V5*, the early regulation of Axin in Wg-responding cells, as revealed by the position, width and timing of appearance of the Axin stripes, was the same in embryos expressing either *attP40 Axin-V5* or *attP33 Axin-V5* (Fig. 5d-i, compare with Fig. 2d–f and Supplementary Fig. 6a–c). Further, both the initiation and the maintenance of *engrailed* expression were similar in embryos expressing either *attP33 Axin-V5* or *attP40 Axin-V5*, as was the embryonic hatch rate (Fig. 5c,j,k, compare with Fig. 4a,b). These results were in sharp contrast with the absence of early Axin stripes, the disrupted *engrailed* expression, and the decreased embryonic hatch rate in embryos expressing *AxinΔTBD* or in *Tnks* mutants expressing *attP33 Axin-V5* (Figs 3g–i,m–o and 4c–f; Supplementary Fig. 12l–n; Fig. 5c). Altogether, these findings demonstrate that the level to which Axin is increased in *Tnks* mutant embryos expressing *Axin-V5* is not sufficient to disrupt Axin regulation or to inhibit Wg signalling. Instead, these results suggested that in addition to its known role in Axin proteolysis, Tnks also acts through a distinct mechanism to promote the initial regulation of Axin and the activation of signalling following Wg exposure.

### Wnt stimulation increases the ADP-ribosylated Axin level

Our unanticipated findings suggested that Tnks-mediated ADP-ribosylation of Axin facilitates rapid Axin regulation and the activation of signalling following Wnt exposure. To test this hypothesis, we examined whether Wnt stimulation modulates the levels of ADP-ribosylated Axin. Detection of ADP-ribosylated Axin has been challenging, as the levels are very low under basal conditions^52^, and ADP-ribosylation does not markedly alter the electrophoretic mobility of Axin^28^,^52^. Therefore, to detect ADP-ribosylated Axin, we utilized a previously developed pull down assay based on the Trp–Trp–Glu (WWE) domain of the RING-type E3 ubiquitin ligase RNF146/Iduna coupled to glutathione *S*-transferase (GST)^52^. Tnks and RNF146 are known to form a stable complex that targets substrates for ADP-ribosylation and subsequent ADP-ribose-dependent ubiquitination^52^,^53^,^54^,^55^. The WWE domain of RNF146 interacts directly with the poly(ADP-ribose) in these Tnks substrates to promote their ubiquitination.

**Figure 6:**
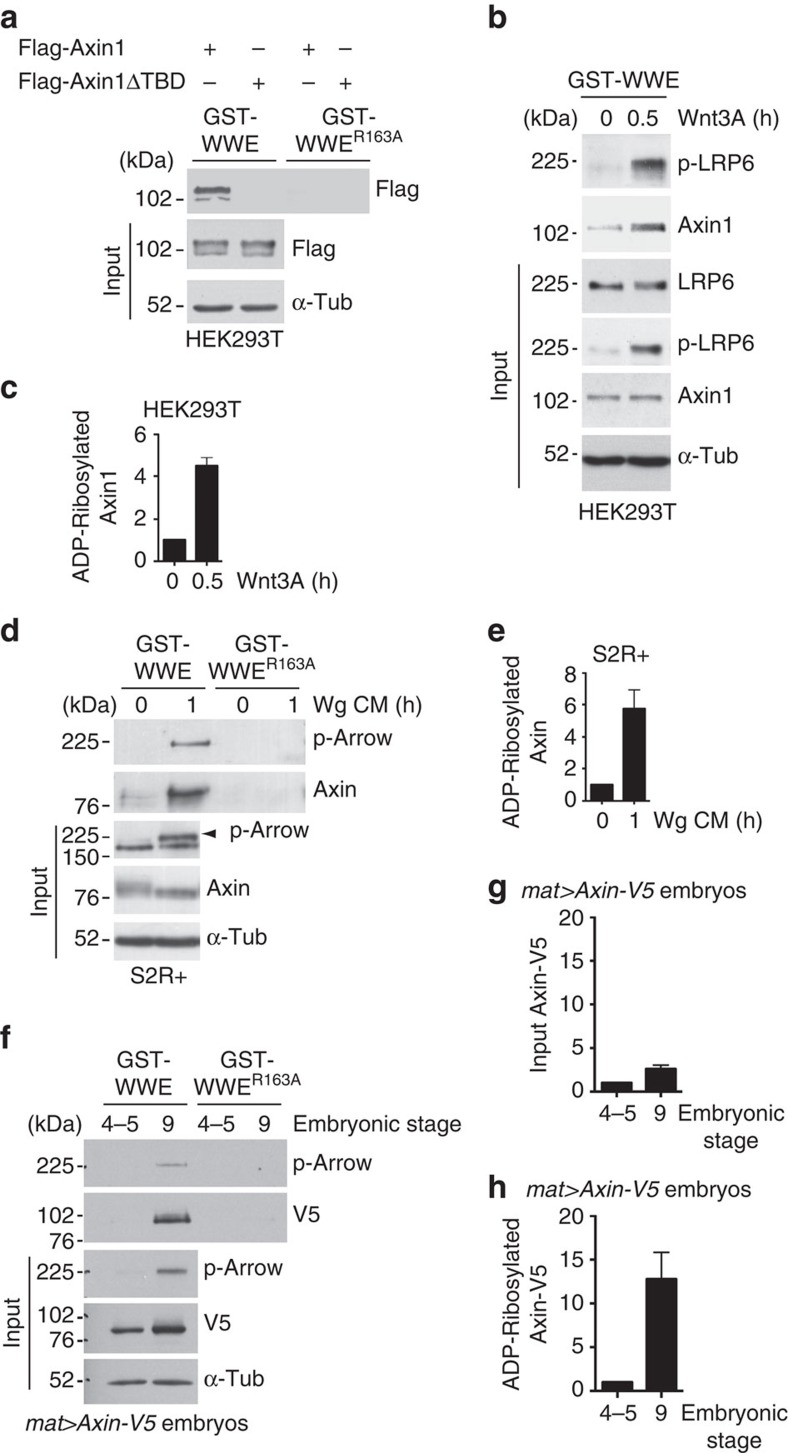
ADP-ribosylated Axin rapidly accumulates following Wnt stimulation. (**a**) Use of a GST-WWE pull down assay for specific detection of ADP-ribosylated Axin. Detection of ADP-ribosylated mouse Flag-Axin1 and Flag-Axin1ΔTBD using either GST-WWE or GST-WWE^R163A^ control pull downs in HEK293T cells. Flag-Axin1 is pulled down by GST-WWE; however, the Flag-Axin1ΔTBD control is not. (**b**,**c**) The levels of ADP-ribosylated Axin rapidly increased following Wnt exposure in human cells. (**b**) HEK293T cells were treated with Wnt3A for 0.5 h, and cell lysates were subsequently pulled down by GST-WWE followed by immunoblot analysis with the indicated antibodies. Following treatment with Wnt, phospho-LRP6 is pulled down by GST-WWE. (**c**) Quantification of relative ADP-ribosylated Axin1 levels from experiments shown in (**b**). Error bars represent s.e.m. of three independent experiments. *P*=0.0062 (Student's *t*-test). (**d**,**e**) The level of ADP-ribosylated Axin rapidly increased following Wg exposure in *Drosophila* cells. (**d**) Immunoblot of lysates from S2R+ cells treated with Wg conditioned medium (CM) for 1 h and then subjected to GST-WWE pull down. Treatment with Wg CM increased the level of ADP-ribosylated Axin pulled down with GST-WWE. Following treatment with Wg CM, phospho-Arrow is pulled down by GST-WWE. (**d**, input) Treatment with Wg CM induced a mobility shift in Axin. (**e**) Quantification of relative levels of ADP-ribosylated Axin from experiments shown in (**d**). Error bars represent s.e.m. of four independent experiments. *P*=0.0147 (Student's *t*-test). (**f**–**h**) The levels of Axin-V5 and ADP-ribosylated Axin-V5 are rapidly increased after the onset of Wg stimulation in *Drosophila* embryos. Embryos at stages 4–5 and stage 9 expressing *Axin-V5* driven by the *mat-Gal4* driver. Stages 4-5 (1.5 to 2.5 h of development) are before the onset of Wg stimulation and stage 9 (3.5 to 4.5 h of development) is after the onset of Wg stimulation. Lysates were immunoblotted by indicated antibodies, followed by GST-WWE pull down. Axin-V5 and phospho-Arrow are pulled down by GST-WWE following Wg stimulation. (**g** and **h**) Quantification of relative Axin-V5 and ADP-ribosylated Axin-V5 levels from experiments shown in (**f**). (**g**) *P*=0.0321 (Student's *t*-test). (**h**) *P*=0.0305 (Student's *t*-test). Error bars represent s.e.m. of three independent experiments.

As a control for specificity of the GST-WWE assay, pull downs were simultaneously performed using a GST-WWE^R164A^ mutant control, in which an arginine to alanine substitution in the RNF146 WWE domain abolishes interaction with poly(ADP-ribose)^52^. As an additional control for specificity, we generated a construct that encodes mouse Axin1 with a nine amino-acid deletion in the TBD, *Axin1ΔTBD*, which is predicted to prevent the Tnks-dependent ADP-ribosylation of Axin1 (refs 28, 47, 48). GST-WWE pull downs were performed with lysates from HEK293T cells expressing either *Flag-Axin1* or *Flag-Axin1ΔTBD*, followed by immunoblotting with Flag antibody (Fig. 6a). Although the levels of Axin1 and Axin1ΔTBD in these lysates were comparable, GST-WWE pulled down Axin1, but not Axin1ΔTBD. Further, neither Axin1 nor Axin1ΔTBD was pulled down by the GST-WWE^R164A^ control. Tnks-mediated ADP-ribosylation does not markedly alter the electrophoretic mobility of Axin^28^,^52^; nonetheless, we detected small mobility shifts in the ADP-ribosylated Axin isolated by GST-WWE pull down (Supplementary Fig. 16a), suggesting that Axin is pulled down through its ADP-ribosylation, rather than through association with another ADP-ribosylated protein. These findings confirmed the specificity of the GST-WWE pull-down assay for the detection of ADP-ribosylated Axin.

To determine whether Wnt stimulation induces changes in the level of endogenous ADP-ribosylated Axin, we performed GST-WWE pull downs on lysates from HEK293T cells treated with Wnt3A protein. The accumulation of phospho-LRP6 was used to monitor activation of the Wnt pathway (Fig. 6b, input). Unexpectedly, within 30 min of Wnt3A exposure, the level of ADP-ribosylated Axin1 increased by four- to fivefold (Fig. 6b,c). These results demonstrate that the pool of ADP-ribosylated Axin increases rapidly following Wnt exposure.

To determine whether the increase in the pool of ADP-ribosylated Axin in response to Wnt exposure is an evolutionarily conserved process, we used GST-WWE pull downs to analyse lysates from *Drosophila* S2R+ cells treated with Wg conditioned medium (Fig. 6d). We analysed two downstream signalling events to monitor the temporal response of these cells to Wg exposure: the phosphorylation of Arrow/LRP6 (refs 8, 56), and the stabilization of Arm/β-catenin^40^,^57^ (Supplementary Fig. 8d). Treatment of S2R+ cells with Wg conditioned medium resulted in increased levels of phospho-Arrow within 10 min, which peaked at 40 to 50 min and remained elevated for more than 2 h. In addition, Arm/β-catenin was stabilized within 20 min of Wg exposure and remained elevated for 2 h (Supplementary Fig. 8d), as reported previously^57^.

**Figure 7:**
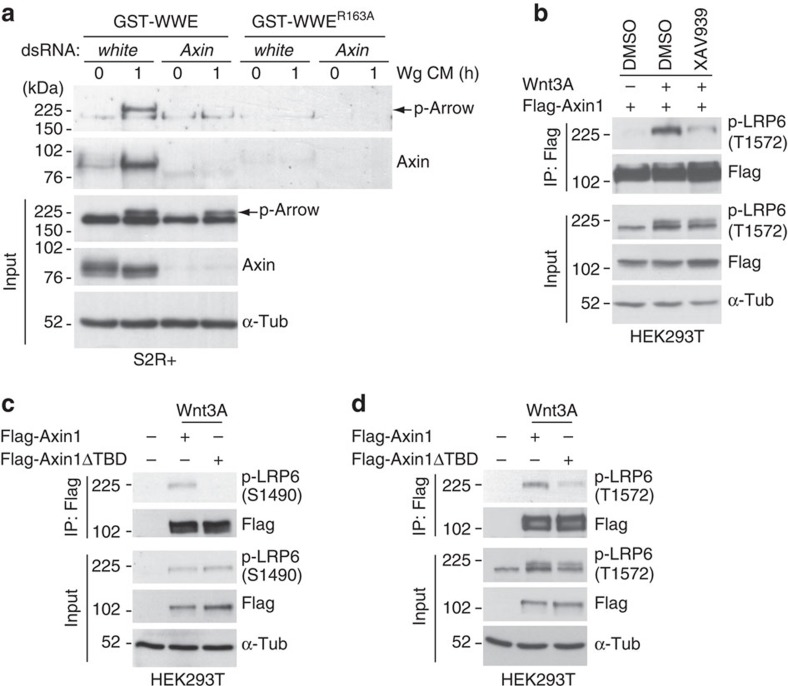
ADP-ribosylation enhances interaction of Axin with phospho-LRP6 following Wnt stimulation. (**a**) Phospho-Arrow interacts with ADP-ribosylated Axin following Wg exposure. Immunoblot of S2R+ cells treated with dsRNA against *Axin* or *white* (negative control), then treated with Wg CM for 1 h, subjected to GST-WWE pull down, and analysed by immunoblotting with indicated antibodies. Treatment with Wg CM markedly increases the amount of ADP-ribosylated Axin pulled down with GST-WWE, but not the GST-WWE^R163A^ control. On treatment with Wg CM, phospho-Arrow is also pulled down with GST-WWE, but not the GST-WWE^R163A^ control. The pull down of phosphorylated Arrow by GST-WWE is prevented by treatment with *Axin* dsRNA. (**a**, input) Treatment with Wg CM induced a mobility shift in Axin. There is a small decrease in the level of phospho-LRP6 following *Axin* knockdown. These data suggest that in Drosophila, Axin is not essential for Wg-dependent phosphorylation of LRP6, but may facilitate this process. (**b**) ADP-ribosylation enhances the interaction of Axin with phospho-LRP6. HEK293T cells expressing *Flag-Axin1* were treated with either the DMSO control or the small molecule Tnks inhibitor XAV939 for 24 h. XAV939 diminishes the Wnt3A-dependent interaction between Axin1 and phospho-LRP6. (**c**,**d**) The Tnks-binding domain enhances the interaction of Axin with phospho-LRP6 following Wnt exposure. HEK293T cells transfected with either Flag-Axin1 or Flag-Axin1ΔTBD, and subsequently treated with Wnt3A for 30 min. Lysates were immunoprecipitated by Flag antibody, followed by immunoblot using indicated antibodies. Both (**c**) phospho-LRP6 (S1490) and (**d**) phospho-LRP6 (T1572) antibodies demonstrate that Axin1 interacts with phospho-LRP6 following Wnt3A stimulation; however, deletion of the TBD of Axin1 diminishes this interaction. α-Tub was used as a loading control. These experiments were performed at least three times with representative results shown.

Our new Axin antibody allowed sensitive detection of endogenous Axin, and revealed a mobility shift following Wg stimulation (Figs 6d and 7a, input), consistent with the Wnt-dependent dephosphorylation of Axin observed previously in mammalian cells^14^,^17^,^18^. GST-WWE pull down followed by immunoblotting with the Axin antibody revealed that the levels of ADP-ribosylated Axin increased within one hour of stimulation with Wg. In contrast, Axin was not pulled down by the GST-WWE^R164A^ control, confirming specificity of the signal (Fig. 6d). The increase in ADP-ribosylated Axin levels ranged from four- to sevenfold, indicating that, as observed in human cells, ADP-ribosylated Axin accumulated rapidly following Wg stimulation in *Drosophila* cells (Fig. 6d,e).

To further test this hypothesis *in vivo*, we quantitated changes in the levels of ADP-ribosylated Axin following Wg stimulation in lysates from staged *Drosophila* embryos expressing *Axin-V5* using GST-WWE pull downs. These studies revealed that the total levels of Axin-V5 increased by approximately threefold in stage 9 embryos (after Wg stimulation) by comparison with stage 4–5 embryos (before Wg stimulation; Fig. 6f,g). Importantly, the level of ADP-ribosylated Axin-V5 increased by ∼13-fold after Wg stimulation (Fig. 6f,h). As described above, small mobility shifts were detected in ADP-ribosylated Axin (Supplementary Fig. 16b). These results indicated that the pool of ADP-ribosylated Axin is preferentially increased following Wg exposure. Therefore, we conclude that the rapid increase in ADP-ribosylated Axin is an evolutionarily conserved response to Wnt/Wg stimulation.

### ADP-ribosylation enhances the interaction of Axin with LRP6

On the basis of the rapid accumulation of ADP-ribosylated Axin in both *Drosophila* and human cells following Wnt exposure, we speculated that ADP-ribosylation of Axin might facilitate Axin's interaction with phospho-LRP6 and the subsequent activation of the Wnt pathway. Indeed, we observed that in lysates of human cells exposed to Wnt, not only Axin, but also phospho-LRP6 was pulled down by GST-WWE (Fig. 6b). Analogously, in lysates of *Drosophila* cultured cells or embryos exposed to Wg, phospho-Arrow was pulled down by GST-WWE, but not the GST-WWE^R164A^ control, indicating a requirement for ADP-ribosylation (Fig. 6d,f). Thus, we hypothesized that both phospho-LRP6 and phospho-Arrow were pulled down through their association with ADP-ribosylated Axin following Wnt exposure. To test this hypothesis, we repeated the GST-WWE pull downs after depleting *Drosophila* Axin using RNAi-mediated knockdown in cultured *Drosophila* cells (Fig. 7a). Treatment of S2R+ cells with *Axin* dsRNA resulted in a marked reduction in Axin levels, to the extent that Arm/β-catenin was aberrantly stabilized in the absence of Wg stimulation (Supplementary Fig. 3i). In the presence or absence of *Axin* knockdown, the level of phospho-Arrow was comparable (Fig. 7a, input); however, the pull down of phospho-Arrow by GST-WWE was completely dependent on Axin (Fig. 7a). Although we cannot exclude the possibility that phospho-LRP6/Arrow is ADP-ribosylated in an Axin-dependent manner, these findings support the hypothesis that following Wg exposure, phospho-Arrow interacts with ADP-ribosylated Axin.

To further test the hypothesis that the ADP-ribosylation of Axin promotes its interaction with phospho-LRP6 following Wnt stimulation, we treated HEK293T cells expressing Flag-Axin1 with either a dimethyl sulfoxide (DMSO) control or with the small molecular inhibitor XAV939 (ref. 28), to inhibit the catalytic activity of the two human Tnks proteins. We immunoprecipitated from lysates of these cells with Flag antibody, followed by immunoblotting with phospho-LRP6 antibodies. Treatment with XAV939, but not the DMSO control, markedly decreased the Wnt-dependent interaction between Axin1 and phospho-LRP6 within 30 min of Wnt3A exposure (Fig. 7b). To further test this hypothesis, we immunoprecipitated lysates of HEK293T cells expressing *Flag-Axin1* or *Flag-Axin1ΔTBD* with Flag antibody, followed by immunoblotting with phospho-LRP6 antibodies directed against either phospho-serine^S1490^ [S1490 (ref. 8)] or phosphothreonine^1572^ [T1572 (ref. 44)]. By 30 min after treatment with Wnt3A, Axin1 interacted with phospho-LRP6, as revealed by both phospho-LRP6 antibodies (Fig. 7c,d)^8^,^12^. In contrast, deletion of the TBD in Axin1 diminished the interaction of Axin with phospho-LRP6 (Fig. 7c,d). Analogously, deletion of the TBD in *Drosophila* Axin decreased the Wg-dependent interaction between Axin and phospho-Arrow (Supplementary Fig. 17a). Deletion of the TBD in human Axin1 had no detectable effect on Axin1's interaction with GSK3β, β-catenin or Dvl (Supplementary Fig. 17b,c). Altogether, these results indicated that the Tnks-dependent ADP-ribosylation of Axin promotes Axin's rapid interaction with phospho-LRP6 following Wnt stimulation.

**Figure 8:**
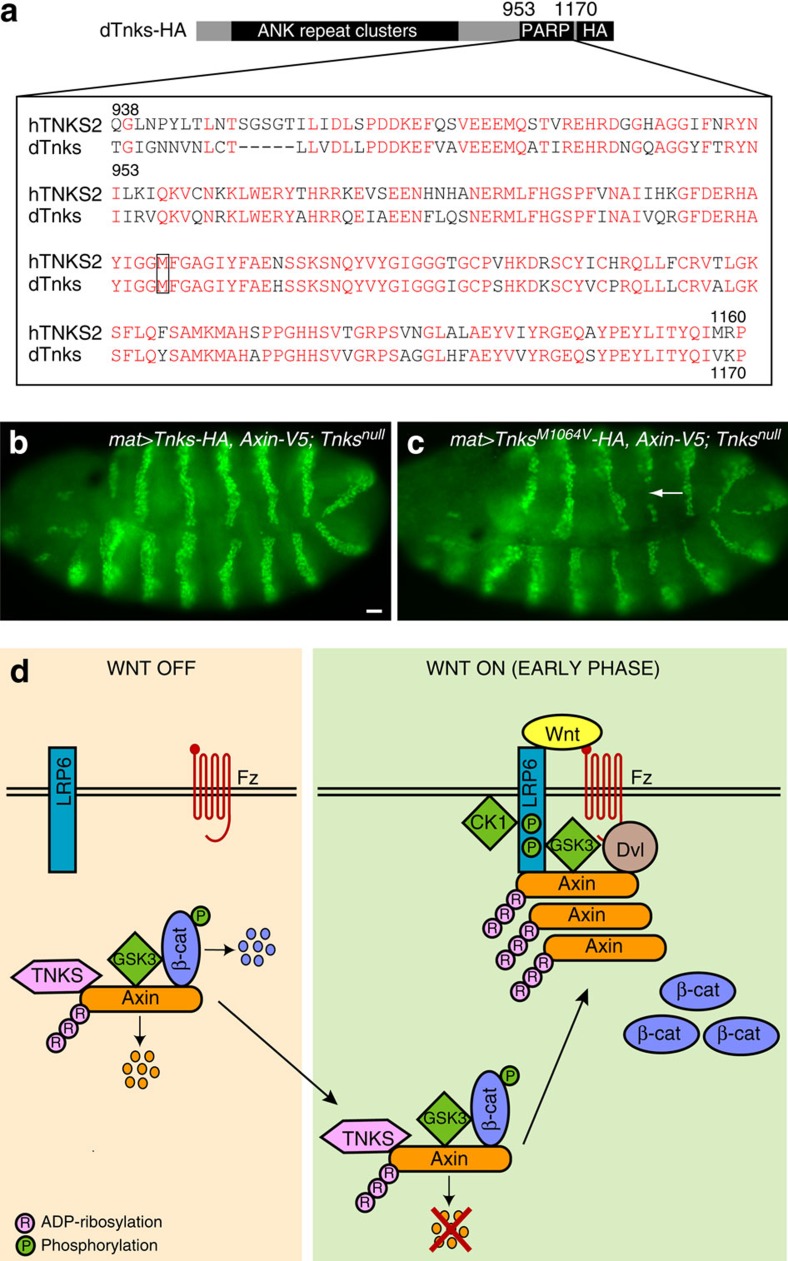
Tnks catalytic activity promotes activation of the Wg pathway. (**a**) Schematic representation of HA-tagged *Drosophila* Tnks (*dTnks-HA*). Alignment below shows amino-acid conservation in the catalytic PARP domain of human and *Drosophila* Tnks. Identical amino acids are in red. Box indicates the substitution in *dTnks*^*M1064V*^ that is predicted to inactivate PARP activity. (**b**,**c**) Wg signalling in the *Tnks* null mutant is rescued by expression of wild-type Tnks, but not catalytically inactive Tnks. Stage 9 embryos stained with En antibody. (**b**) In *Tnks* null mutant embryos expressing *Tnks-HA* and *Axin-V5* driven by the *mat-Gal4* driver, the width of the En stripes is normal (2–3 cells in width). (**c**) In *Tnks* null mutant embryos expressing *Tnks*^*M1064V*^*-HA and Axin-V5* driven by the *mat-Gal4* driver, En stripes are aberrantly narrowed (arrow). Scale bar, 25 μm. (**d**) Model for the dual roles of Tnks in Wnt signalling. Tnks-dependent ADP-ribosylation not only targets Axin for proteolysis independently of Wnt stimulation, but also promotes signalling immediately following Wnt exposure. Wnt stimulation induces the rapid accumulation of ADP-ribosylated Axin. ADP-ribosylation promotes Axin's interaction with the Wnt co-receptor LRP6, thereby activating the Wnt pathway.

Finally, we tested whether the poly(ADP-ribosyl) polymerase (PARP) activity of Tnks promotes the initial activation of Wnt signalling. We generated HA-tagged *Tnks* transgenes for expression in *Drosophila* embryos: one transgene encoded wild-type Tnks, whereas the other transgene was identical, except for a methionine to valine substitution (M1064V) in the catalytic PARP domain (Fig. 8a). The analogous mutation (M1054V) in the highly conserved PARP domain of human Tnks2 is known to abolish its catalytic activity^58^. Both *Tnks* transgenes were integrated at the same genomic site, and expressed using the *mat-Gal4* driver. As noted above, in *Tnks* null mutant embryos expressing *Axin-V5*, the Wg-dependent expression of *engrailed* was disrupted (Fig. 4f). In contrast, *engrailed* expression was fully restored in *Tnks* mutant embryos expressing the wild-type *Tnks* transgene (Fig. 8b), but not the *Tnks*^*M1064V*^ transgene (Fig. 8c). Together, these findings support the conclusion that the ADP-ribosylation of Axin by Tnks promotes not only the interaction between Axin and phospho-LRP6 following Wg exposure, but also the initial activation of Wg signalling.

## Discussion

We demonstrate that Wnt exposure induces biphasic regulation in the level of Axin, and a large increase in the level of ADP-ribosylated Axin immediately after stimulation. ADP-ribosylation enhances the interaction of Axin with phospho-LRP6, and promotes the activation of Wnt signalling. Our findings lead to three major revisions of the current model for the role of Tnks in the activation of the Wnt pathway. First, Tnks serves bifunctional roles under basal conditions and after stimulation, revealing a remarkable economy and coordination of pathway components. Second, our results provide a mechanistic basis for the rapid reprogramming of Axin function in response to Wnt stimulation, and thereby reveal an unanticipated role for Tnks in this process (Fig. 8d). These findings suggest that Wnt exposure either rapidly increases the ADP-ribosylation of Axin or inhibits the targeting of ADP-ribosylated Axin for proteasomal degradation, through mechanisms yet to be elucidated. Finally, we demonstrate that pharmacologic inactivation of Tnks diminishes the interaction of Axin with LRP6, revealing a previously unknown mechanism through which small molecule Tnks inhibitors disrupt Wnt signalling, distinct from their known role in stabilizing the destruction complex.

In the absence of Wnt stimulation, the concentration-limiting levels of Axin regulate its scaffold function in the destruction complex^15^,^16^. As components of the destruction complex participate in other signalling pathways, the low levels of Axin were proposed to maintain modularity of the Wnt pathway^16^. Our new findings indicate that Axin levels are not only regulated in the absence of Wnt, but also regulated biphasically following Wnt stimulation. This sequential modulation of Axin divides activation of the pathway into an early, fast phase and a delayed long-term phase. During embryogenesis, the earliest expression of Wg triggers the rapid appearance of Axin in segmental stripes, which is a novel hallmark for the initial activation of the pathway. Our findings reveal that Wnt exposure induces a rapid increase in the total level of Axin, and importantly, a preferential increase in the level of the ADP-ribosylated Axin. The early Axin stripes are absent in *Tnks* null mutant embryos and are also absent when the Tnks binding domain in Axin is deleted. Therefore, we propose that Axin ADP-ribosylation contributes to Axin stabilization and to the rapid response to Wg stimulation.

We postulate that the initial increase in levels of ADP-ribosylated Axin jump-starts the response to Wnt stimulation by enhancing the Axin-LRP6 interaction, whereas the subsequent decrease in Axin levels prolongs the duration of signalling by reducing destruction complex assembly. Thus, Wnt stimulation induces rapid increases in the levels of not only cytoplasmic β-catenin, but also ADP-ribosylated Axin. Previous work that coupled mathematical modelling with experimental analysis revealed that several Wnt signalling systems were responsive to the relative change in β-catenin levels, rather than their absolute value^59^. This dependence was proposed to impart robustness and resistance to noise and cellular variation. Our data raise the possibility that a similar principle applies to changes in Axin levels on the Axin-LRP6 interaction, as the marked increase in ADP-ribosylated Axin levels following Wnt stimulation is evolutionarily conserved. Thus, the relative change in levels of ADP-ribosylated Axin may promote signalling following Wnt exposure by facilitating the fold change in β-catenin levels.

Our findings have relevance for the context-specific *in vivo* roles of Tnks in Wnt signalling suggested in previous studies. Tnks inhibition disrupts Wnt signalling in a number of cultured cell lines^28^, but *in vivo* studies in several model organisms suggested that the requirement for Tnks in promoting Wnt signalling is restricted to specific cell types or developmental stages. In mice, functional redundancy exists between the two Tnks homologues^60^, such that *Tnks* single mutants are viable and fertile, whereas double mutants display embryonic lethality without overt Wnt-related phenotypes^60^ (see Discussion in ref. 61). However, a missense mutation in the TBD of Axin2 that is predicted to disrupt ADP-ribosylation resulted in either activating or inhibiting effects on Wnt signalling that were dependent on developmental stage. Tnks inhibitors resulted in the same paradoxical effects, suggesting complex roles in mouse embryonic development^61^. Analogously, treatment of fish with Tnks inhibitors resulted in no observed defects in Wnt-mediated processes during development^28^; however, the regeneration of injured fins in adults, a process that requires Wnt signalling^62^, was disrupted^28^,^63^.

Similarly, the finding that *Drosophila Tnks* null mutants are viable^49^,^50^,^51^ was unexpected, as Tnks is highly evolutionarily conserved, and no other Tnks homologues exist in fly genomes. Nonetheless, our studies reveal that a less than twofold increase in Axin levels uncovers the importance of Tnks in promoting Wg signalling during embryogenesis. Therefore, we postulate that Tnks loss can be compensated during development unless Axin levels are increased, but that the inhibition of Wg signalling resulting from Tnks inactivation cannot be attributed solely to increased Axin levels (Supplementary Fig. 3, Figs 4f and 5). Furthermore, *Drosophila* Tnks is essential for Wg target gene activation in the adult intestine, and exclusively within regions of the gradient where Wg is present at relatively low concentration^49^,^64^. Thus, the context-specific roles of Tnks observed in different model organisms may reflect the mechanisms described herein, which reveal that the Wnt-induced association of Axin with LRP6 occurs even in the absence of Axin ADP-ribosylation, but is markedly enhanced in its presence. We postulate that by enhancing this interaction, Tnks-dependent ADP-ribosylation of Axin serves to amplify the initial response to Wnt stimulation, and thus is essential in a subset of *in vivo* contexts.

The recent discovery that Tnks enhances signalling in Wnt-driven cancers has raised the possibility that Tnks inhibitors will offer a promising new therapeutic option^28^,^63^. Indeed, preclinical studies have supported this possibility^30^. Tnks inhibitors were thought previously to disrupt Wnt signalling solely by increasing the basal levels of Axin, and thus by increasing destruction complex activity. However, our findings indicate that the degree to which the basal level of Axin increases following Tnks inactivation is not sufficient to disrupt Wnt signalling in some *in vivo* contexts. Instead, our results reveal that Tnks inhibition simultaneously disrupts signalling at two critical and functionally distinct steps: by promoting activity of the destruction complex and by diminishing an important step in signalosome assembly: the Wnt-induced interaction between LRP6 and Axin. On the basis of these findings, we propose that the efficacy of Tnks inhibitors results from their combined action at both of these steps, providing a rationale for their use in the treatment of a broad range of Wnt-driven cancers. Therefore, our results suggest that in contrast with the current focus on tumours in which attenuation of the destruction complex aberrantly activates Wnt signalling (such as those lacking APC), the preclinical testing of Tnks inhibitors could be expanded to include cancers that are dependent on pathway activation by Wnt stimulation. These include the colorectal, gastric, ovarian and pancreatic cancers that harbour inactivating mutations in *RNF43* (refs 65, 66, 67, 68, 69, 70), a negative Wnt feedback regulator that promotes degradation of the Wnt co-receptors Frizzled and LRP6 (refs 70, 71, 72).

## Methods

### *Drosophila* stocks and genetics

To generate the *pUASTattB Axin-V5* transgene, we isolated a KpnI/HindIII fragment from *pAc5.1-Daxin-3xHA* (ref. 28) and a HindIII/XbaI fragment encoding a triple V5 epitope from *pBlue SK-3xV5* (ref. 73), and ligated these fragments into *pUASTattB* (refs 36, 37), at the KpnI and XbaI sites. To generate the *pUASTattB-AxinΔTBD-V5* transgene, residues D-12 through K-32 were deleted by PCR-based mutagenesis of *pUASTattB-Axin-V5* using the oligonucleotide: 5′-GGTATCTGCTACCCCTTCGGTCATATGTTTCCGGATTCC-3′. The resulting *AxinΔTBD-V5* fragment was digested with KpnI and XbaI, and inserted into the *pUASTattB vector* at the KpnI and XbaI sites. Transgenic flies were generated using phiC31-based integration^36^ at the *attP33* or *attP40* sites on chromosome 2 (ref. 35).

*pUASTattB Axin-GFP* was generated using the Gateway LR Clonase system (Invitrogen), with an entry plasmid containing an *Axin* cDNA amplified by PCR from an ovarian library^74^ and the *pTWG* destination vector containing sequences encoding *GFP* (Drosophila Genomics Resource Center). *Axin-GFP* was amplified by PCR using primers that introduced KpnI and XbaI restriction sites at the 5′ and 3′ ends, respectively. Following KpnI and XbaI digestion, the amplified product was cloned into *pUASTattB*. Transgenic flies were generated using phiC31-based site-specific integration at the *attP33 site*. *Axin-GFP* has an 18 amino-acid linker sequence between the carboxy terminus of Axin and the amino terminus of GFP: KPSD/kgradpaflykvvssat/(GFP) (‘/' represents the junction between Axin and GFP, with additional amino acids shown in lowercase). The *pUASTattB-AxinΔTBD-GFP* transgene was generated by PCR-based mutagenesis of *pUASTattB-Axin-GFP*, in which residues D-12 through K-32 were deleted using the oligonucleotide: 5′-GGAATCCGGAAACATATGACCGAAGGGGTAGCAGATACC-3′. The resulting *AxinΔTBD-GFP* fragment was digested with KpnI and XbaI and inserted into the *pUASTattB* vector at the KpnI and XbaI sites.

To generate the *pUASTattB Tnks-HA* transgene, a PCR fragment encoding Tnks was amplified from the cDNA *LD22548* (Drosophila Genomics Research Center), with the addition of an XhoI restriction site to 5′ end using the primer 5′-ACTCTCGAGATGGCCAACAGCAGCCGAAGT-3′. A fragment encoding a double-HA epitope followed by a KpnI restriction site was added to the 3′ end of the *Tnks* cDNA using the PCR primer 5′-CATAGTCCGGGACGTCATAGGGATAGCCCGCATAGTCAGGAACATC-3′. The *Tnks-HA* fragment was digested with XhoI and KpnI, and inserted into the *pUASTattB* vector at the XhoI and KpnI sites. *pUASTattB Tnks*^*M1064V*^*-HA* was generated by replacing the methionine at position 1,064 with valine by PCR-based mutagenesis of *pUAST-Tnks-2* × *HA* using the oligonucleotide 5′-ATTGGCGGCGTGTTTGGGGCT-3′, and cloned into the XhoI and KpnI restriction sites of *pUASTattB.* Transgenic flies were generated using phiC31-based integration at the *attP40* site.

To obtain embryos expressing *Axin-V5* driven by *mat-Gal4*: *mat-Gal4* females were crossed to *UAS-Axin-V5* males. F1 female progeny of the genotype *UAS-Axin-V5 / mat-Gal4* were crossed to *UAS-Axin-V5* males.

To obtain *Tnks* null mutant embryos expressing *Axin-V5* driven by *mat-Gal4*: *UAS-Axin-V5; Tnks*^*19*^ *mutant* males were crossed to *mat-Gal4; Tnks*^*19*^ females. F1 female progeny of the genotype *UAS-Axin-V5/mat-Gal4; Tnks*^*19*^ were crossed to *UAS-Axin-V5*; *Tnks*^*19*^ males.

To obtain *Tnks* null mutant embryos expressing *Tnks-HA* and *Axin-V5* driven by *mat-Gal4*: *UAS-Tnks-HA*, *UAS-Axin-V5*; *Tnks*^*503*^ mutant males were crossed to *mat-Gal4; Tnks*^*503*^ females. F1 female progeny of the genotype *UAS-Tnks-HA*, *UAS-Axin-V5/mat-Gal4*; *Tnks*^*503*^ were crossed to *UAS-Tnks-HA*, *UAS-Axin-V5*; *Tnks*^*503*^ males.

To obtain Tnks null mutant embryos expressing *Tnks^M1064V^-HA* and *Axin-V5* driven by *mat-Gal4: UAS-Tnks^M1064V^-HA, UAS-Axin-V5; Tnks^503^* mutant males were crossed to *mat-Gal4; Tnks^503^* females. F1 female progeny of the genotype *UAS-Tnks^M1064V^-HA, UAS-Axin-V5/mat-Gal4; Tnks^503^* were crossed to *UAS-Tnks^M1064V^-HA, UAS-Axin-V5; Tnks^503^* males.

### Antibodies

The primary antibodies used for immunostaining were rabbit anti-V5 (1:1,000, Abcam, ab9116), rabbit anti-GFP (1:200, Invitrogen, A11122), mouse anti-Engrailed (1:100, concentrated 4D9; Developmental Studies Hybridoma Bank, DSHB), mouse anti-Wg (1:200, 4D4 concentrated antibody, DSHB), mouse anti-Arm (1:20, N2 7A1, DSHB) and mouse anti-Neurotactin (1:20, BP106, DSHB). Secondary antibodies were goat or donkey Alexa Fluor 488 or 555 conjugates (1:400, Invitrogen, A21202, A11008, A31572, A31570). The primary antibodies used for immunoblotting were mouse anti-V5 (1:5,000, Invitrogen, 46-1157), guinea pig anti-Axin (1:1,000 (ref. 49)), mouse anti-Wg (1:100, 4D4, DSHB), rabbit anti-Kinesin Heavy Chain (1:10,000, Cytoskeleton, AKIN01), mouse anti-Arm (1:100, N2 7A1, DSHB), mouse anti-α-Tubulin (1:10,000, DM1A, Sigma, T6199), rabbit anti-α-Tubulin (1:10,000, Sigma, SAB3501071), mouse anti-Flag M2 (1:1000, Sigma, F3165), rabbit anti-GSK3β (1:1,000, Cell Signaling, 9315), mouse anti-β-catenin (1:4,000, BD Biosciences, 610154), rabbit anti-Axin1 (1:1,000, Cell Signaling, #2074), rabbit anti-LRP6 (1:1,000, Cell Signaling, 2560), rabbit anti-c-Myc (1:1,000, Santa Cruz Biotechnology, sc-40), rabbit anti-phospho-LRP6 [Ser1490] (1:1,000, Cell Signaling^8^, 2568), rabbit anti-phospho-LRP6 [Thr1572] (1:1,000, Millipore^44^, 07-2187), and guinea pig anti-Arrow (1:1,000 (ref. 75)). Secondary antibodies used for immunoblotting were goat anti-rabbit HRP conjugate (1:10,000, Biorad, 170-6515), goat anti-mouse HRP conjugate (1:10,000, Biorad, 170-6516) and goat anti-guinea pig HRP conjugate (1:10,000, Jackson ImmunoResearch).

### GST-WWE pull downs

For GST pull downs, GST-WWE and GST-WWE^R164A^ beads were generated as described previously^52^. Embryos, S2R+ or HEK293T cells were treated as indicated, then washed once with cold 1 × PBS and lysed in lysis buffer (50 mM Tris-HCl [pH 8.0], 100 mM NaCl, 1% NP-40, 10% glycerol, 1.5 mM EDTA [pH 8.0]) or RIPA buffer (50 mM Tris-HCl (pH 8.5), 300 mM NaCl, 1% NP-40, 0.5% sodium deoxycholate and 0.1% SDS) supplemented with 1 μM of the poly(ADP-ribose) glycohydrolase inhibitor ADP-HDP (Enzo Life Sciences), and protease and phosphatase inhibitor cocktail (1:100, Thermo Scientific). Lysates were incubated with GST-WWE or GST-WWE^R164A^ beads overnight at 4 °C. Following incubation, beads were washed four times in wash buffer (50 mM Tris-HCl [pH 8.0], 150 mM NaCl, 1% NP-40, 10% Glycerol, 1.5 mM EDTA [pH 8.0]) supplemented with 1 μM ADP-HPD and protease and phosphatase inhibitor cocktail (1:100). Bound materials were eluted with 2 × sample buffer and resolved by SDS–PAGE, transferred to PVDF or nitrocellulose membranes and blotted with the indicated antibodies.

### Plasmids

Plasmids used for transfection of human cell lines were *pCS2+ Flag-mouse Axin1* (ref. 8) and *pCS2+ Flag-mouse Axin1ΔTBD*. To generate the *pCS2+ Flag-mouse Axin1ΔTBD* plasmid, residues P-26 through E-34 were deleted by PCR-based mutagenesis of *pCS2+ Flag-mouse Axin1* using the oligonucleotide: 5′-GATGCCGGAGAACTGGTATCTACTGAT-3′. The resulting Flag-mouse *AxinΔTBD* fragment was digested with ClaI and BglII, and then inserted into the *pCS2+ vector* at the ClaI and BglII sites.

Plasmids used for transfection of *Drosophila* cells were *pAc5.1-Axin-V5* and *pAc5.1-AxinΔTBD-V5*. To generate the *pAc5.1-Axin-V5* and *pAc5.1-AxinΔTBD-V5* plasmids: fragments encoding Axin-V5 and AxinΔTBD-V5 from *pUASTattB-Axin-V5* and *pUASTattB-AxinΔTBD-V5*, respectively, were digested using KpnI and XbaI. The resulting fragments were inserted into the *pAc5.1* vector (Invitrogen) at the KpnI and XbaI sites.

### Study design and statistical anaysis

In all experiments reported in this study, Student's *t*-test and ANOVA test were performed using Prism (GraphPad Software Inc., CA, USA) or the SAS version 9.4 (Statistical Analysis System Institute Inc., NC, USA). Student's *t*-test was used to compare two groups for all data sets. ANOVA test was used for comparison of more than two groups. *P* values and detailed test statistics are provided in the figure legends. No blinding was done and no particular randomization method was used. All experiments were performed at least three times.

Additional methods and fly stocks used are described in Supplementary Information.

## Additional information

**How to cite this article:** Yang, E. *et al*. Wnt pathway activation by ADP-ribosylation. *Nat. Commun.* 7:11430 doi: 10.1038/ncomms11430 (2016).

## Supplementary Material

Supplementary Information Supplementary Figures 1-18, Supplementary Methods and Supplementary References

## References

[b1] MacDonald B. T., Tamai K. & He X. Wnt/beta-catenin signaling: components, mechanisms, and diseases. Dev. Cell. 17, 9–26 (2009). 19619488 10.1016/j.devcel.2009.06.016 PMC2861485

[b2] Clevers H. & Nusse R. Wnt/beta-catenin signaling and disease. Cell 149, 1192–1205 (2012). 22682243 10.1016/j.cell.2012.05.012

[b3] Ikeda S. . Axin, a negative regulator of the Wnt signaling pathway, forms a complex with GSK-3beta and beta-catenin and promotes GSK-3beta-dependent phosphorylation of beta-catenin. EMBO J. 17, 1371–1384 (1998). 9482734 10.1093/emboj/17.5.1371 PMC1170485

[b4] Behrens J. . Functional interaction of an axin homolog, conductin, with beta-catenin, APC, and GSK3beta. Science 280, 596–599 (1998). 9554852 10.1126/science.280.5363.596

[b5] Kimelman D. & Xu W. beta-catenin destruction complex: insights and questions from a structural perspective. Oncogene 25, 7482–7491 (2006). 17143292 10.1038/sj.onc.1210055

[b6] Stamos J. L. & Weis W. I. The beta-catenin destruction complex. Cold Spring Harb. Perspect. Biol. 5, a007898 (2013). 23169527 10.1101/cshperspect.a007898 PMC3579403

[b7] Mao J. . Low-density lipoprotein receptor-related protein-5 binds to Axin and regulates the canonical Wnt signaling pathway. Mol. Cell. 7, 801–809 (2001). 11336703 10.1016/s1097-2765(01)00224-6

[b8] Tamai K. . A mechanism for Wnt coreceptor activation. Mol. Cell. 13, 149–156 (2004). 14731402 10.1016/s1097-2765(03)00484-2

[b9] Bilic J. . Wnt induces LRP6 signalosomes and promotes dishevelled-dependent LRP6 phosphorylation. Science 316, 1619–1622 (2007). 17569865 10.1126/science.1137065

[b10] Zeng X. . A dual-kinase mechanism for Wnt co-receptor phosphorylation and activation. Nature 438, 873–877 (2005). 16341017 10.1038/nature04185 PMC2100418

[b11] Davidson G. . Casein kinase 1gamma couples Wnt receptor activation to cytoplasmic signal transduction. Nature 438, 867–872 (2005). 16341016 10.1038/nature04170

[b12] Zeng X. . Initiation of Wnt signaling: control of Wnt coreceptor Lrp6 phosphorylation/activation via frizzled, dishevelled and axin functions. Development 135, 367–375 (2008). 18077588 10.1242/dev.013540 PMC5328672

[b13] Schwarz-Romond T., Metcalfe C. & Bienz M. Dynamic recruitment of axin by Dishevelled protein assemblies. J. Cell. Sci. 120, 2402–2412 (2007). 17606995 10.1242/jcs.002956

[b14] Kim S. E. . Wnt stabilization of beta-catenin reveals principles for morphogen receptor-scaffold assemblies. Science 340, 867–870 (2013). 23579495 10.1126/science.1232389 PMC3788643

[b15] Salic A., Lee E., Mayer L. & Kirschner M. W. Control of beta-catenin stability: reconstitution of the cytoplasmic steps of the wnt pathway in Xenopus egg extracts. Mol. Cell. 5, 523–532 (2000). 10882137 10.1016/s1097-2765(00)80446-3

[b16] Lee E., Salic A., Kruger R., Heinrich R. & Kirschner M. W. The roles of APC and Axin derived from experimental and theoretical analysis of the Wnt pathway. PLoS Biol. 1, E10 (2003). 14551908 10.1371/journal.pbio.0000010 PMC212691

[b17] Luo W. . Protein phosphatase 1 regulates assembly and function of the beta-catenin degradation complex. EMBO J. 26, 1511–1521 (2007). 17318175 10.1038/sj.emboj.7601607 PMC1829374

[b18] Willert K., Shibamoto S. & Nusse R. Wnt-induced dephosphorylation of axin releases beta-catenin from the axin complex. Genes Dev. 13, 1768–1773 (1999). 10421629 10.1101/gad.13.14.1768 PMC316878

[b19] Li V. S. . Wnt signaling through inhibition of beta-catenin degradation in an intact Axin1 complex. Cell 149, 1245–1256 (2012). 22682247 10.1016/j.cell.2012.05.002

[b20] Cselenyi C. S. . LRP6 transduces a canonical Wnt signal independently of Axin degradation by inhibiting GSK3's phosphorylation of beta-catenin. Proc. Natl Acad. Sci. USA 105, 8032–8037 (2008). 18509060 10.1073/pnas.0803025105 PMC2430354

[b21] Kofron M. . Wnt11/beta-catenin signaling in both oocytes and early embryos acts through LRP6-mediated regulation of axin. Development 134, 503–513 (2007). 17202189 10.1242/dev.02739

[b22] Liu X., Rubin J. S. & Kimmel A. R. Rapid, Wnt-induced changes in GSK3beta associations that regulate beta-catenin stabilization are mediated by Galpha proteins. Curr. Biol. 15, 1989–1997 (2005). 16303557 10.1016/j.cub.2005.10.050

[b23] Tolwinski N. S. . Wg/Wnt signal can be transmitted through arrow/LRP5,6 and Axin independently of Zw3/Gsk3beta activity. Dev. Cell. 4, 407–418 (2003). 12636921 10.1016/s1534-5807(03)00063-7

[b24] Yamamoto H. . Phosphorylation of axin, a Wnt signal negative regulator, by glycogen synthase kinase-3beta regulates its stability. J. Biol. Chem. 274, 10681–10684 (1999). 10196136 10.1074/jbc.274.16.10681

[b25] Valvezan A. J., Zhang F., Diehl J. A. & Klein P. S. Adenomatous polyposis coli (APC) regulates multiple signaling pathways by enhancing glycogen synthase kinase-3 (GSK-3) activity. J. Biol. Chem. 287, 3823–3832 (2012). 22184111 10.1074/jbc.M111.323337 PMC3281685

[b26] Malbon C. C. & Wang H. Y. Dishevelled: a mobile scaffold catalyzing development. Curr. Top. Dev. Biol. 72, 153–166 (2006). 16564334 10.1016/S0070-2153(05)72002-0

[b27] Hernandez A. R., Klein A. M. & Kirschner M. W. Kinetic Responses of beta-Catenin Specify the Sites of Wnt Control. Science 338, 1337–1340 (2012). 23138978 10.1126/science.1228734

[b28] Huang S. M. . Tankyrase inhibition stabilizes axin and antagonizes Wnt signalling. Nature 461, 614–620 (2009). 19759537 10.1038/nature08356

[b29] Waaler J. . A novel tankyrase inhibitor decreases canonical Wnt signaling in colon carcinoma cells and reduces tumor growth in conditional APC mutant mice. Cancer Res. 72, 2822–2832 (2012). 22440753 10.1158/0008-5472.CAN-11-3336

[b30] Lau T. . A novel tankyrase small-molecule inhibitor suppresses APC mutation-driven colorectal tumor growth. Cancer Res. 73, 3132–3144 (2013). 23539443 10.1158/0008-5472.CAN-12-4562

[b31] Liu C. & He X. Destruction of a destructor: a new avenue for cancer therapeutics targeting the Wnt pathway. J. Mol. Cell Biol. 2, 70–73 (2010). 20008332 10.1093/jmcb/mjp040 PMC2861491

[b32] van den Heuvel M., Nusse R., Johnston P. & Lawrence P. A. Distribution of the wingless gene product in Drosophila embryos: a protein involved in cell-cell communication. Cell 59, 739–749 (1989). 2582493 10.1016/0092-8674(89)90020-2

[b33] Baker N. E. Molecular cloning of sequences from wingless, a segment polarity gene in Drosophila: the spatial distribution of a transcript in embryos. EMBO J. 6, 1765–1773 (1987). 16453776 10.1002/j.1460-2075.1987.tb02429.x PMC553553

[b34] Cliffe A., Hamada F. & Bienz M. A role of Dishevelled in relocating Axin to the plasma membrane during wingless signaling. Curr. Biol. 13, 960–966 (2003). 12781135 10.1016/s0960-9822(03)00370-1

[b35] Markstein M., Pitsouli C., Villalta C., Celniker S. E. & Perrimon N. Exploiting position effects and the gypsy retrovirus insulator to engineer precisely expressed transgenes. Nat. Genet. 40, 476–483 (2008). 18311141 10.1038/ng.101 PMC2330261

[b36] Bischof J., Maeda R. K., Hediger M., Karch F. & Basler K. An optimized transgenesis system for Drosophila using germ-line-specific phiC31 integrases. Proc. Natl Acad. Sci. USA 104, 3312–3317 (2007). 17360644 10.1073/pnas.0611511104 PMC1805588

[b37] Brand A. H. & Perrimon N. Targeted gene expression as a means of altering cell fates and generating dominant phenotypes. Development 118, 401–415 (1993). 8223268 10.1242/dev.118.2.401

[b38] Hacker U. & Perrimon N. DRhoGEF2 encodes a member of the Dbl family of oncogenes and controls cell shape changes during gastrulation in Drosophila. Genes Dev. 12, 274–284 (1998). 9436986 10.1101/gad.12.2.274 PMC316438

[b39] Hamada F. . Negative regulation of Wingless signaling by D-axin, a Drosophila homolog of axin. Science 283, 1739–1742 (1999). 10073940 10.1126/science.283.5408.1739

[b40] Riggleman B., Schedl P. & Wieschaus E. Spatial expression of the Drosophila segment polarity gene armadillo is posttranscriptionally regulated by wingless. Cell 63, 549–560 (1990). 2225066 10.1016/0092-8674(90)90451-j

[b41] Bejsovec A. & Martinez Arias A. Roles of wingless in patterning the larval epidermis of Drosophila. Development 113, 471–485 (1991). 1782860 10.1242/dev.113.2.471

[b42] DiNardo S., Sher E., Heemskerk-Jongens J., Kassis J. A. & O'Farrell P. H. Two-tiered regulation of spatially patterned engrailed gene expression during Drosophila embryogenesis. Nature 332, 604–609 (1988). 3282172 10.1038/332604a0 PMC2753417

[b43] Nusslein-Volhard C. & Wieschaus E. Mutations affecting segment number and polarity in Drosophila. Nature 287, 795–801 (1980). 6776413 10.1038/287795a0

[b44] MacDonald B. T., Yokota C., Tamai K., Zeng X. & He X. Wnt signal amplification via activity, cooperativity, and regulation of multiple intracellular PPPSP motifs in the Wnt co-receptor LRP6. J. Biol. Chem. 283, 16115–16123 (2008). 18362152 10.1074/jbc.M800327200 PMC2414294

[b45] Leung J. Y. . Activation of AXIN2 expression by beta-catenin-T cell factor. A feedback repressor pathway regulating Wnt signaling. J. Biol. Chem. 277, 21657–21665 (2002). 11940574 10.1074/jbc.M200139200

[b46] Jho E. H. . Wnt/beta-catenin/Tcf signaling induces the transcription of Axin2, a negative regulator of the signaling pathway. Mol. Cell. Biol. 22, 1172–1183 (2002). 11809808 10.1128/MCB.22.4.1172-1183.2002 PMC134648

[b47] Morrone S., Cheng Z., Moon R. T., Cong F. & Xu W. Crystal structure of a Tankyrase-Axin complex and its implications for Axin turnover and Tankyrase substrate recruitment. Proc. Natl Acad. Sci. USA 109, 1500–1505 (2012). 22307604 10.1073/pnas.1116618109 PMC3277157

[b48] Guettler S. . Structural basis and sequence rules for substrate recognition by Tankyrase explain the basis for cherubism disease. Cell 147, 1340–1354 (2011). 22153077 10.1016/j.cell.2011.10.046

[b49] Wang Z. . The ADP-ribose polymerase Tankyrase regulates adult intestinal stem cell proliferation during homeostasis in Drosophila. Development (in the press). 10.1242/dev.127647 PMC4874480 27190037

[b50] Wang Z. . Wnt/Wingless pathway activation is promoted by a critical threshold of Axin maintained by tumor supressor Apc and ADP-ribose polymerase Tankyrase. Genetics 203, 1–13 (2016). 10.1534/genetics.115.183244 PMC4858779 26975665

[b51] Feng Y. . The Drosophila tankyrase regulates Wg signaling depending on the concentration of Daxin. Cell Signal. 26, 1717–1724 (2014). 24768997 10.1016/j.cellsig.2014.04.014 PMC4346149

[b52] Zhang Y. . RNF146 is a poly(ADP-ribose)-directed E3 ligase that regulates axin degradation and Wnt signalling. Nat. Cell. Biol. 13, 623–629 (2011). 21478859 10.1038/ncb2222

[b53] DaRosa P. A. . Allosteric activation of the RNF146 ubiquitin ligase by a poly(ADP-ribosyl)ation signal. Nature 517, 223–226 (2014). 25327252 10.1038/nature13826 PMC4289021

[b54] Callow M. G. . Ubiquitin ligase RNF146 regulates tankyrase and Axin to promote Wnt signaling. PLoS ONE 6, e22595 (2011). 21799911 10.1371/journal.pone.0022595 PMC3143158

[b55] Wang Z. . Recognition of the iso-ADP-ribose moiety in poly(ADP-ribose) by WWE domains suggests a general mechanism for poly(ADP-ribosyl)ation-dependent ubiquitination. Genes Dev. 26, 235–240 (2012). 22267412 10.1101/gad.182618.111 PMC3278890

[b56] Wehrli M. . arrow encodes an LDL-receptor-related protein essential for Wingless signalling. Nature 407, 527–530 (2000). 11029006 10.1038/35035110

[b57] Yanagawa S., Lee J. S. & Ishimoto A. Identification and characterization of a novel line of Drosophila Schneider S2 cells that respond to wingless signaling. J. Biol. Chem. 273, 32353–32359 (1998). 9822716 10.1074/jbc.273.48.32353

[b58] Sbodio J. I., Lodish H. F. & Chi N. W. Tankyrase-2 oligomerizes with tankyrase-1 and binds to both TRF1 (telomere-repeat-binding factor 1) and IRAP (insulin-responsive aminopeptidase). Biochem. J. 361, 451–459 (2002). 11802774 10.1042/0264-6021:3610451 PMC1222327

[b59] Goentoro L. & Kirschner M. W. Evidence that fold-change, and not absolute level, of beta-catenin dictates Wnt signaling. Mol. Cell. 36, 872–884 (2009). 20005849 10.1016/j.molcel.2009.11.017 PMC2921914

[b60] Chiang Y. J. . Tankyrase 1 and tankyrase 2 are essential but redundant for mouse embryonic development. PLoS ONE 3, e2639 (2008). 18612384 10.1371/journal.pone.0002639 PMC2441437

[b61] Qian L., Mahaffey J. P., Alcorn H. L. & Anderson K. V. Tissue-specific roles of Axin2 in the inhibition and activation of Wnt signaling in the mouse embryo. Proc. Natl Acad. Sci. USA 108, 8692–8697 (2011). 21555575 10.1073/pnas.1100328108 PMC3102376

[b62] Wehner D. & Weidinger G. Signaling networks organizing regenerative growth of the zebrafish fin. Trends Genet. 31, 336–343 (2015). 25929514 10.1016/j.tig.2015.03.012

[b63] Chen B. Z. . Small molecule-mediated disruption of Wnt-dependent signaling in tissue regeneration and cancer. Nat. Chem. Biol. 5, 100–107 (2009). 19125156 10.1038/nchembio.137 PMC2628455

[b64] Tian A., Benchabane H., Wang Z. & Ahmed Y. Regulation of stem cell proliferation and cell fate specification by wingless/Wnt signaling gradients enriched at adult intestinal compartment boundaries. PLoS Genet. 12, e1005822 (2016). 26845150 10.1371/journal.pgen.1005822 PMC4742051

[b65] Jiao Y. . Whole-exome sequencing of pancreatic neoplasms with acinar differentiation. J. Pathol. 232, 428–435 (2014). 24293293 10.1002/path.4310 PMC4048021

[b66] Ryland G. L. . RNF43 is a tumour suppressor gene mutated in mucinous tumours of the ovary. J. Pathol. 229, 469–476 (2013). 23096461 10.1002/path.4134

[b67] Wang K. . Whole-genome sequencing and comprehensive molecular profiling identify new driver mutations in gastric cancer. Nat. Genet. 46, 573–582 (2014). 24816253 10.1038/ng.2983

[b68] Giannakis M. . RNF43 is frequently mutated in colorectal and endometrial cancers. Nat. Genet. 46, 1264–1266 (2014). 25344691 10.1038/ng.3127 PMC4283570

[b69] Ivanov I., Lo K. C., Hawthorn L., Cowell J. K. & Ionov Y. Identifying candidate colon cancer tumor suppressor genes using inhibition of nonsense-mediated mRNA decay in colon cancer cells. Oncogene 26, 2873–2884 (2007). 17086209 10.1038/sj.onc.1210098

[b70] Koo B. K. . Tumour suppressor RNF43 is a stem-cell E3 ligase that induces endocytosis of Wnt receptors. Nature 488, 665–669 (2012). 22895187 10.1038/nature11308

[b71] Hao H. X. . ZNRF3 promotes Wnt receptor turnover in an R-spondin-sensitive manner. Nature 485, 195–200 (2012). 22575959 10.1038/nature11019

[b72] de Lau W., Peng W. C., Gros P. & Clevers H. The R-spondin/Lgr5/Rnf43 module: regulator of Wnt signal strength. Genes Dev. 28, 305–316 (2014). 24532711 10.1101/gad.235473.113 PMC3937510

[b73] Moqtaderi Z. & Struhl K. Expanding the repertoire of plasmids for PCR-mediated epitope tagging in yeast. Yeast 25, 287–292 (2008). 18338317 10.1002/yea.1581

[b74] Grosshans J., Schnorrer F. & Nusslein-Volhard C. Oligomerisation of Tube and Pelle leads to nuclear localisation of dorsal. Mech. Dev. 81, 127–138 (1999). 10330490 10.1016/s0925-4773(98)00236-6

[b75] Marois E., Mahmoud A. & Eaton S. The endocytic pathway and formation of the Wingless morphogen gradient. Development 133, 307–317 (2006). 16354714 10.1242/dev.02197

