# Supplementary material for: Wnt pathway activation by ADP-ribosylation
Source: Nat Commun. 2016 May 3;7:11430. doi: 10.1038/ncomms11430 (PMC4857404; doi:10.1038/ncomms11430)
Supplement: Supplementary Information — Supplementary Figures 1-18, Supplementary Methods and Supplementary References [file ncomms11430-s1.pdf]

Fig. S1.

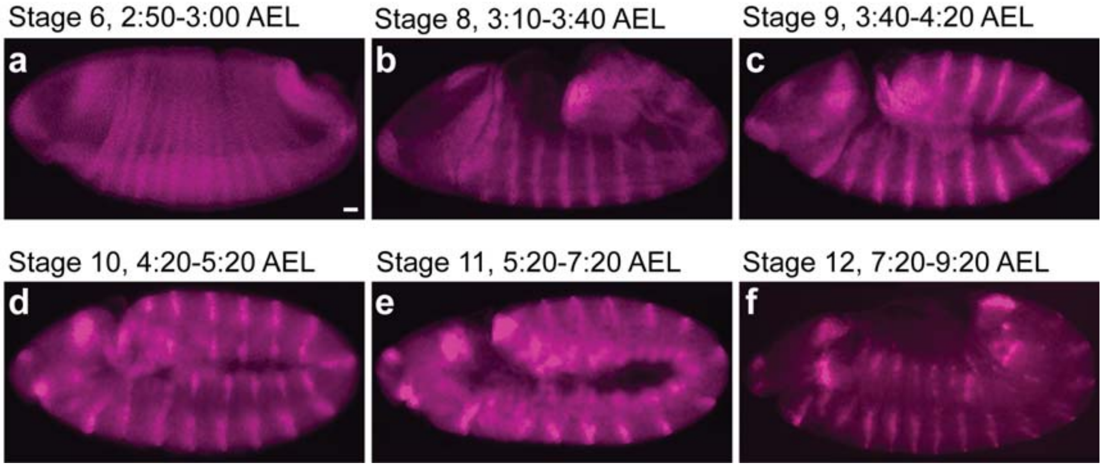

Supplementary Figure 1. Wingless expression in the ectoderm during embryonic development. Confocal images of wild-type embryos stained with Wg antibody. Anterior left, dorsal top. Embryonic stage and hours after egg lay (AEL) are indicated on top. (a) Wg protein is first detectable at stage 6, and observed in weak segmental stripes in the embryonic ectoderm. (b and c) Through stages 8 and 9 [1], Wg stripes intensify. (d-f) By mid-stage 10, Wg expression splits into ventral and dorsal domains, which continues to stage 12. Scale bars represent 25 μm.

Fig. S2.

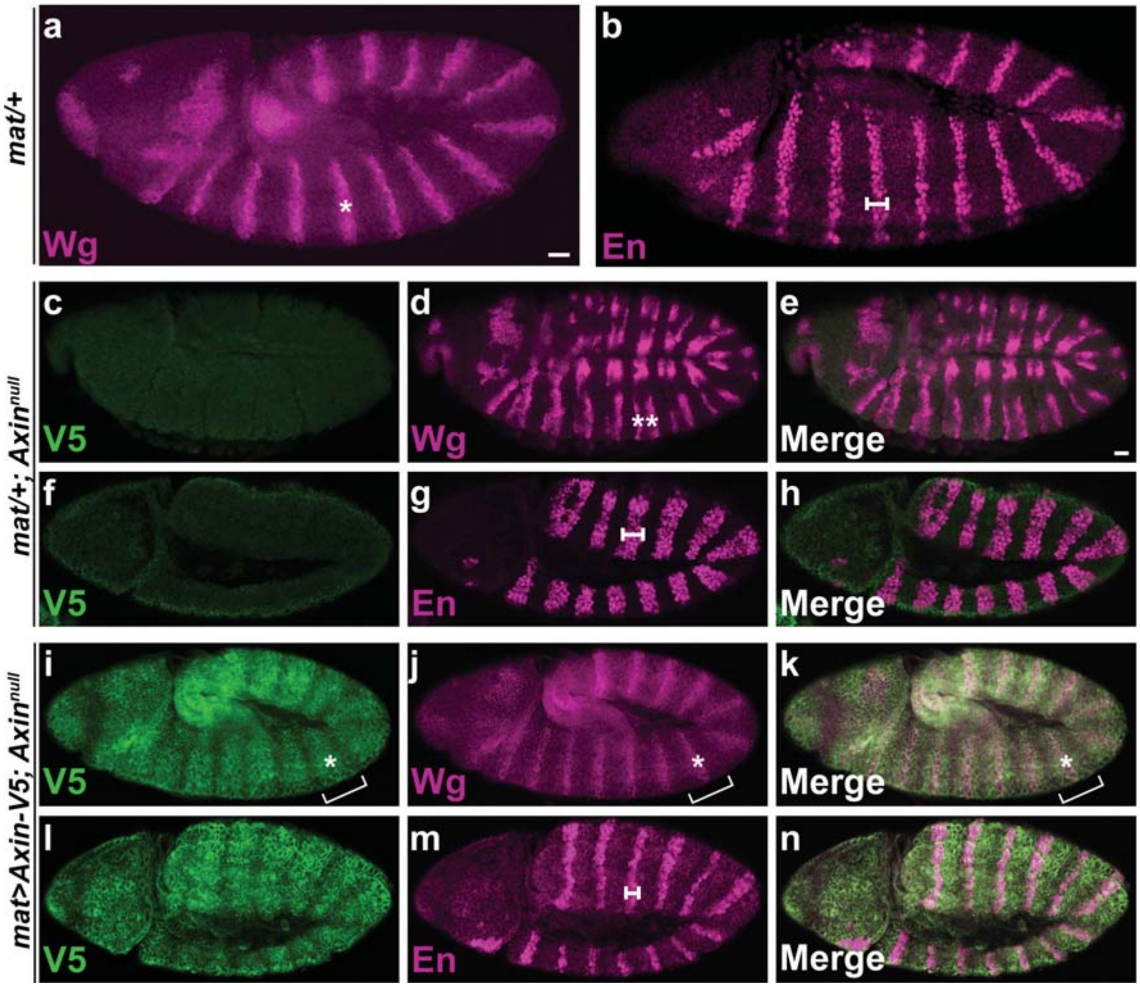

Supplementary Figure 2. *Axin-V5* driven by the *mat-Gal4* driver replaces the function and regulation of endogenous *Axin*. Confocal images of stage 9 embryos stained with V5, En, and Wg antibodies. (a and b) Control embryos have a wild-type number (14) of Wg stripes (a, asterisk), and the normal two to three cell width of En stripes (b, white bar) within each parasegment. (c-h) In maternal/zygotic *Axin*^{s044230} null mutant embryos, ectopic Wg signaling results in a doubling of the Wg stripes (d, asterisks) and expansion in the width of the En stripes (g, white bar). (i-n) Maternal and zygotic *Axin*^{s044230} null mutant embryos at stage 9 expressing *Axin-V5* with the *mat-Gal4*

driver. Axin-V5 is present in segmental stripes (i, bracket) in the embryonic ectoderm that overlap the Wg stripes (k, asterisk). Expression of Axin-V5 in the *Axin* mutant restores the Wg stripes to the normal number (j, asterisk), and restores En stripes to the normal width (m, white bar) within each parasegment. Scale bars represent 25 μm.

Supplementary Figure 3.

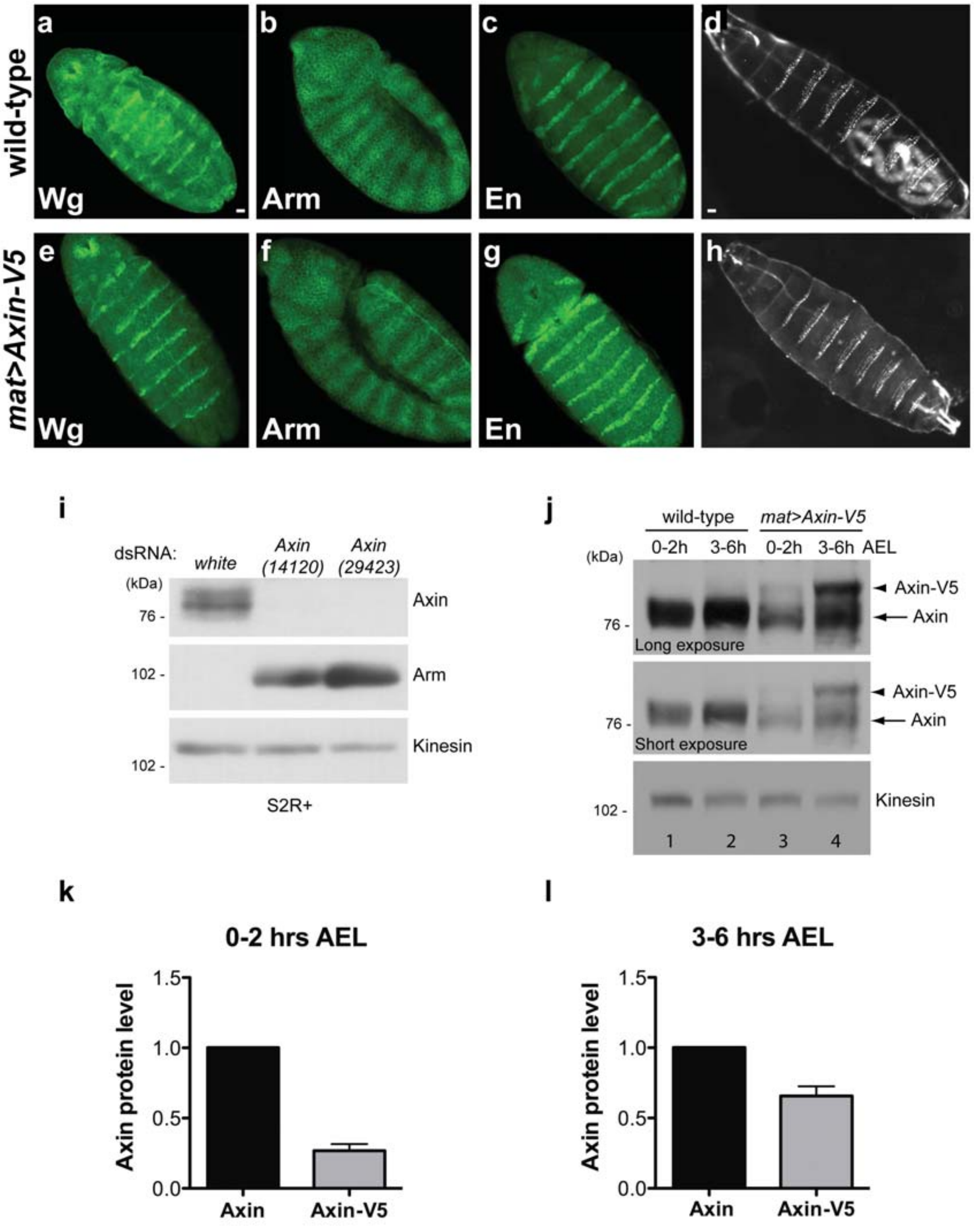

Supplementary Figure 3. Expression of *Axin-V5* in wild-type embryos is compatible with normal Winglessg expression and Winglessg pathway activation. Confocal images of stage 9 or 10 wild-type embryos (a-c) or embryos expressing *Axin-V5* driven by the *mat-Gal4* driver (e-g) stained with Wg, Arm, or En antibodies. Anterior is oriented at the top left corner. (a and e) Normal Wg expression in segmental stripes is observed in both genotypes. (b and f) Arm accumulates in a striped pattern in Wg responding cells in both genotypes. (c and g) En expression is maintained in stripes of 2 to 3 cells in width in response to Wg in both genotypes. (d and h) First instar larval cuticles reveal normal Wg-dependent cell fate specification in both genotypes, as indicated by the presence of naked cuticle that alternates with denticle belts. Scale bars represent 25 μm. (i) Specificity of the Axin antibody. S2R+ cells treated with either dsRNA against *white* (negative control) or two distinct dsRNAs against *Axin* (14120 or 29423). Treatment with *Axin* dsRNA results in reduction of Axin protein and the aberrant stabilization of Arm in the absence of Wg stimulation. (j) Comparison of the levels of endogenous Axin with Axin-V5. Immunoblot analysis of lysates from wild-type embryos or embryos expressing *Axin-V5* driven by the *mat-Gal4* driver. Embryos were collected at 0-2 hours AEL (prior to the onset of Wingless expression) and 3-6 hours AEL (after the onset of Wingless expression). The specificity of the Axin-V5 signal is revealed by its absence in wild-type embryos (lanes 1 and 2). The level of Axin-V5 (arrowhead) is lower than that of endogenous Axin (arrow) at 0-2 hours and 3-6 hours of development (lanes 3 and 4). Kinesin was used as a loading control. Three independent experiments were performed. (k,l) Quantification of experiments shown in (j). The relative levels of Axin and Axin-V5 measured in immunoblots of lysates from embryos expressing *Axin-*

V5 at 0-2 hours AEL (k) and 3-6 hours (l). Error bars represent s.e.m. of three independent experiments. (k) *P*=0.0041 (Student’s *t*-test). (l) *P*=0.0377 (Student’s *t*-test).

Fig. S4.

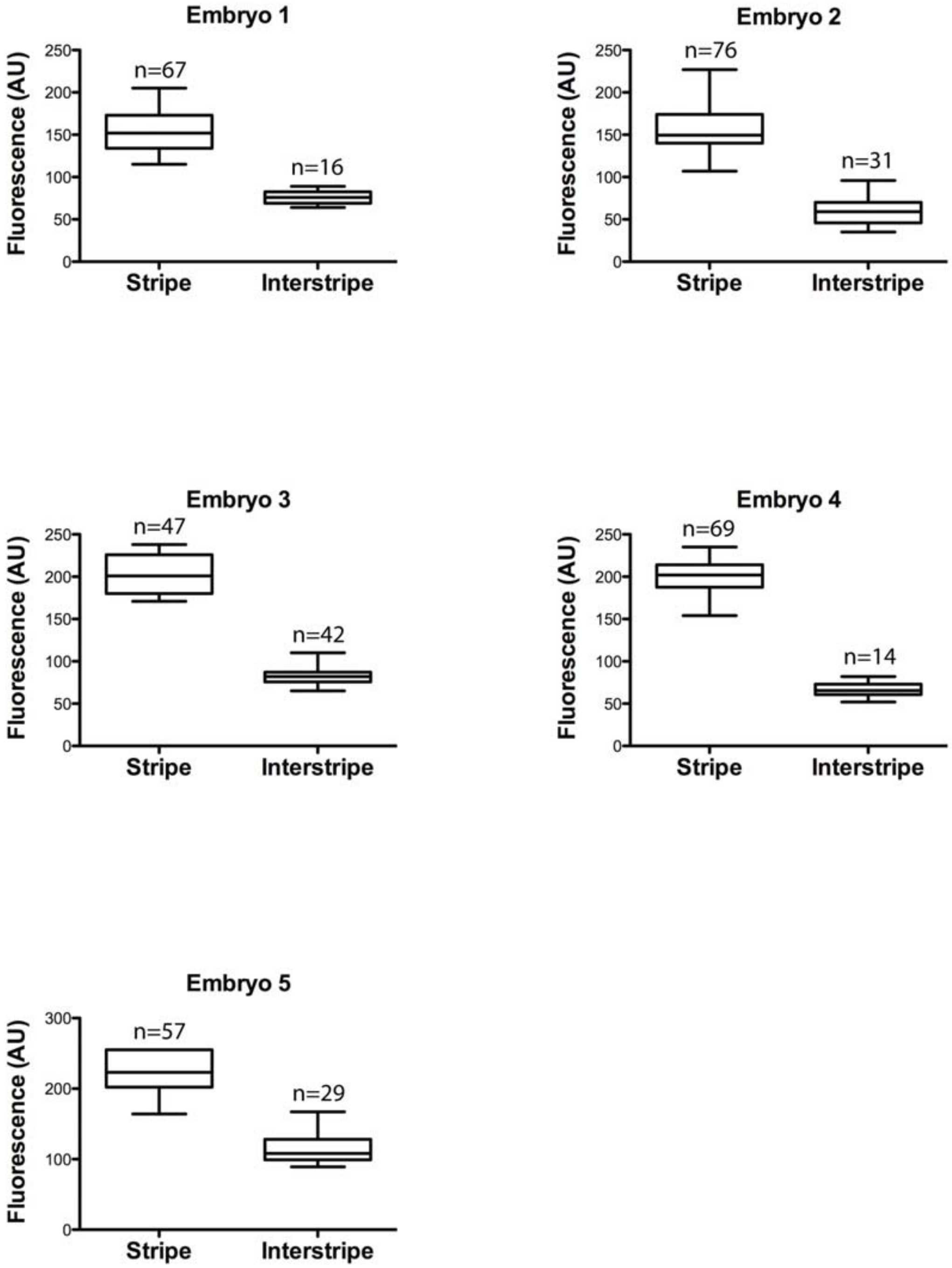

Supplementary Figure 4. Axin levels increase in segmental stripes in the

embryonic ectoderm. Box plot showing the distribution of V5 fluorescence intensity (arbitrary units, AU) within stripes and interstripes in stage 9 embryos expressing *Axin-V5* driven by the *mat-Gat4* driver and stained with V5 antibody. Axin intensity within the stripes is two- to three-fold higher than that in the interstripes. Five embryos were randomly captured from over hundred embryos. Fluorescence microscope settings remained unchanged for the entire experiment. n numbers indicate the data points. The normality assumption was not violated. P<0.0001 (unpaired heteroscedastic *t*-test). Box plot explanation: upper horizontal line of box, 75th percentile; lower horizontal line of box, 25th percentile; horizontal bar within box, median; whiskers, minimum and maximum data point.

Fig. S5.

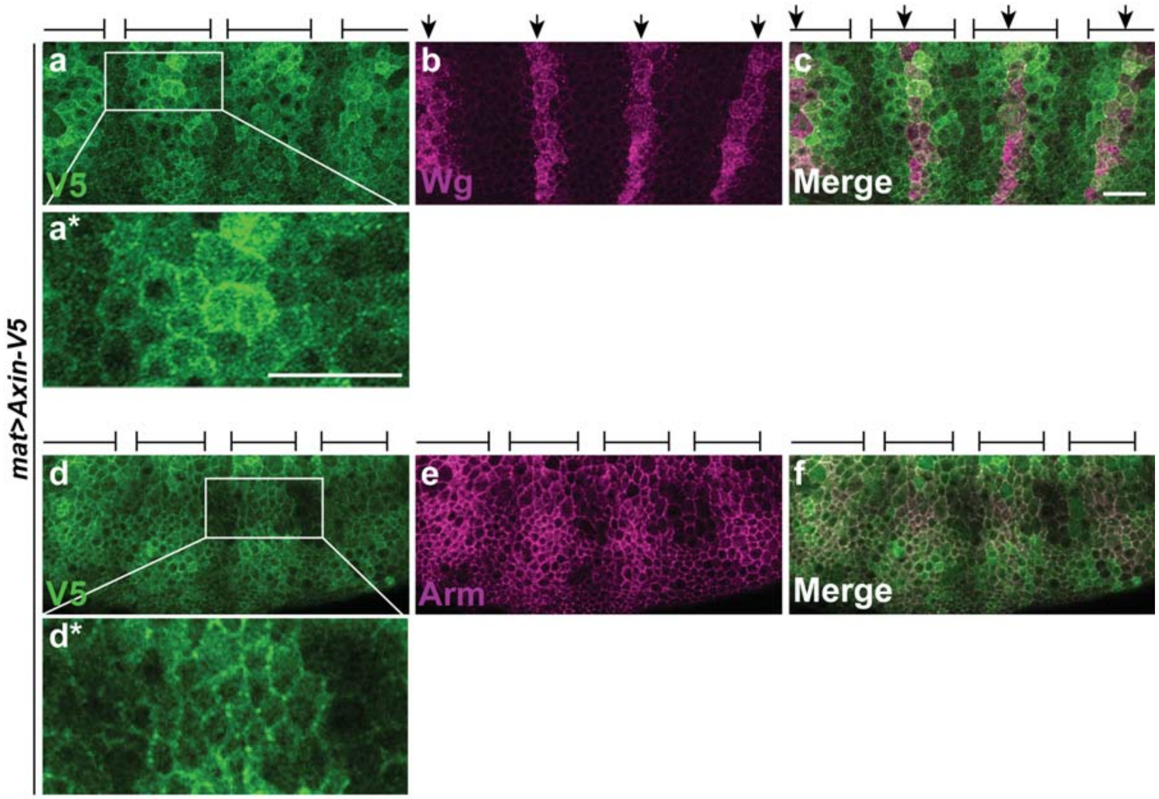

Supplementary Figure 5. The formation of Axin stripes in Wingless responding cells. Confocal images of stage 9 embryos expressing *Axin-V5* driven by *mat-Gal4* driver, stained with V5, Wg, or Arm antibodies. (a-c) Axin-V5 is distributed in wide segmental stripes (brackets on top) that overlap the Wg stripes (arrows on top). (d-f) Axin-V5 stripes co-localize with stripes of stabilized Arm protein (brackets on top). (a* and d*) Higher magnification of corresponding boxed region. Scale bars represent 25 μm.

Fig. S6.

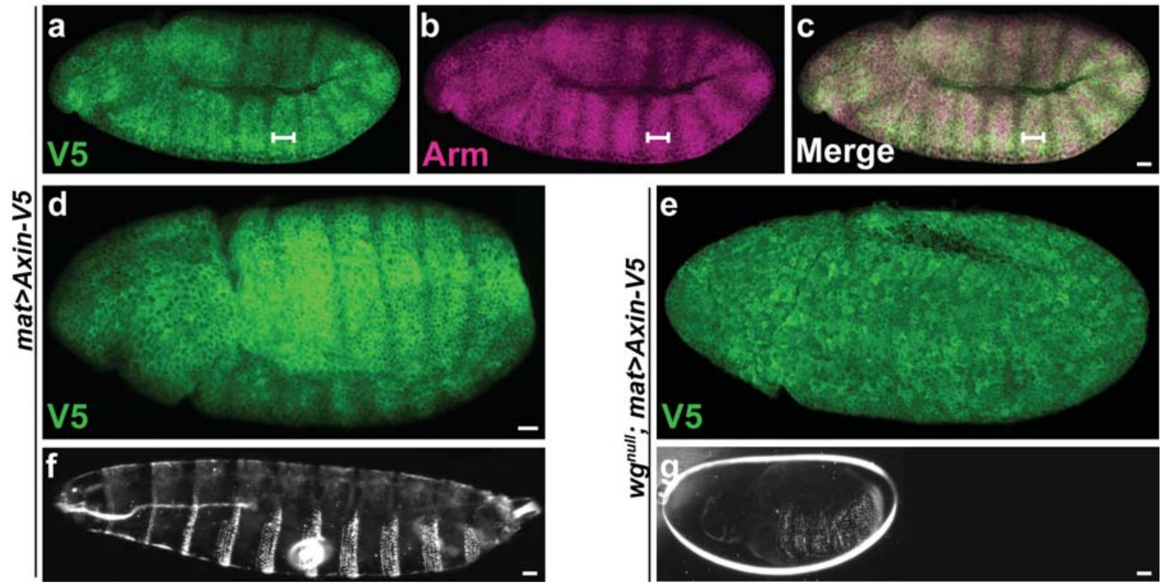

Supplementary Figure 6. Wingless is required for Axin stabilization. (a-c) Confocal images of stage 9 embryos expressing *Axin-V5* driven by the *mat-Gal4* driver. Co-staining with V5 and Arm antibodies shows that Axin-V5 accumulates in cells that are responding to Wg exposure, as evidenced by the accumulation of Arm (bracket). (d and e) Stage 9 embryos expressing *Axin-V5* driven by the *mat-Gal4* driver. The segmental stripes of Axin-V5 present in wild-type embryos (d) are lost in the *wg*^{CX4} null mutant embryos (e). The denticle pattern of first instar larval cuticles reveals proper cell fate specification, indicated by the presence of naked cuticle in each segment of wild-type embryos expressing *Axin-V5* (f), and aberrant cell fate specification in the *wg*^{CX4} null mutant, indicated by the lawn of denticles (g). Scale bars represent 25 μm.

Fig. S7.

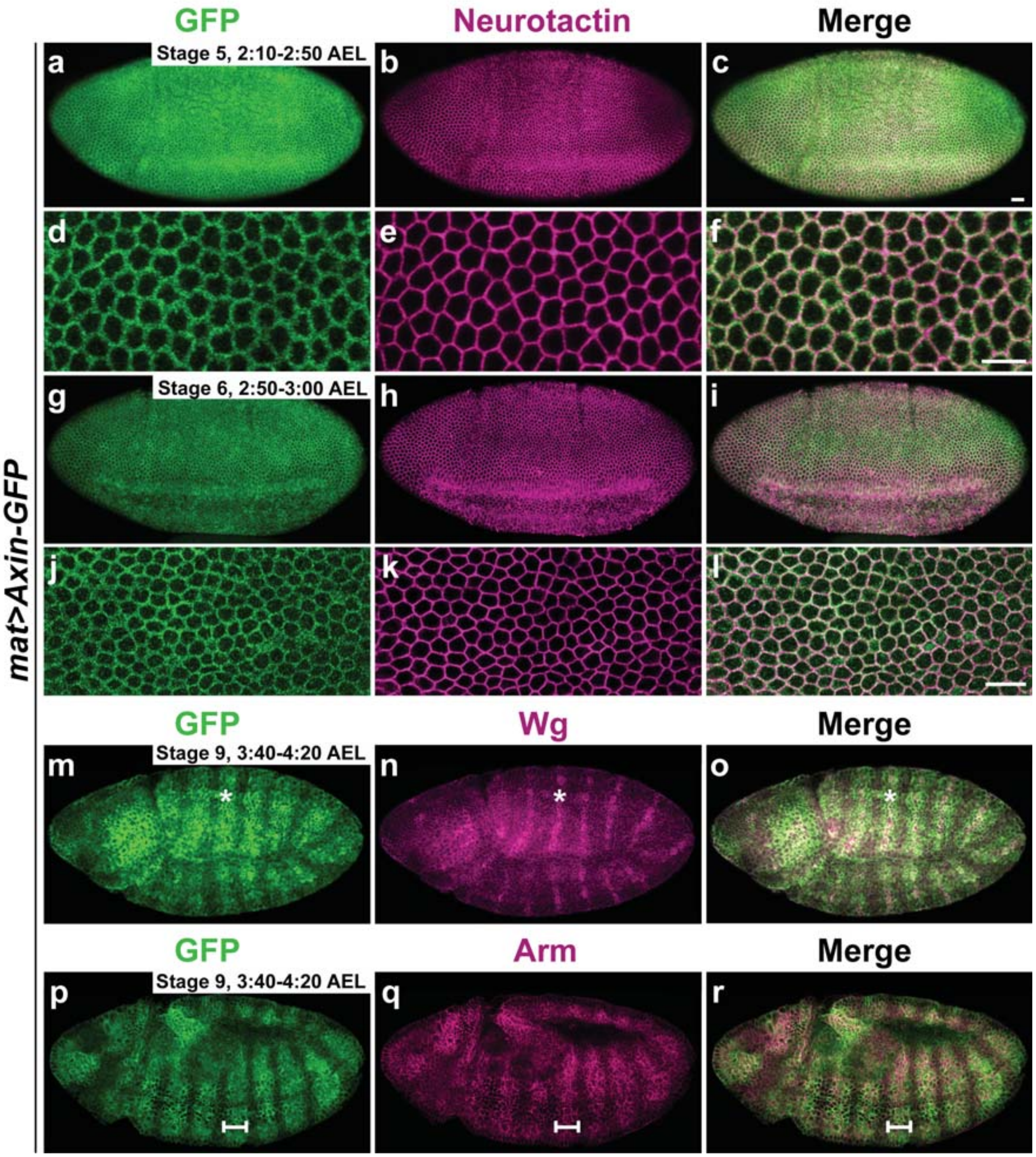

Supplementary Figure 7. Axin-GFP and Axin-V5 are regulated identically prior to, during and after the onset of Wingless expression. Confocal images of embryos expressing *Axin-GFP* driven by the *mat-Gal4* driver. (a-l) In stage 5 and 6 embryos, Axin-GFP is uniformly distributed throughout the ectoderm, as is the transmembrane

protein Neurotactin. (d-f and j-l) Higher magnification images show that Axin-GFP co-localizes with Neurotactin. (m-o) By 30 minutes after the onset of Wg expression (late stage 8 to stage 9), Axin-GFP accumulates in wide segmental stripes of increased staining intensity that overlap the Wg stripes (asterisks). (p-r) Axin-GFP stripes co-localize with stripes of Arm protein (white bars), indicating that Axin intensity is increased in the cells responding to Wg exposure. Scale bars represent 25 μm.

Fig. S8.

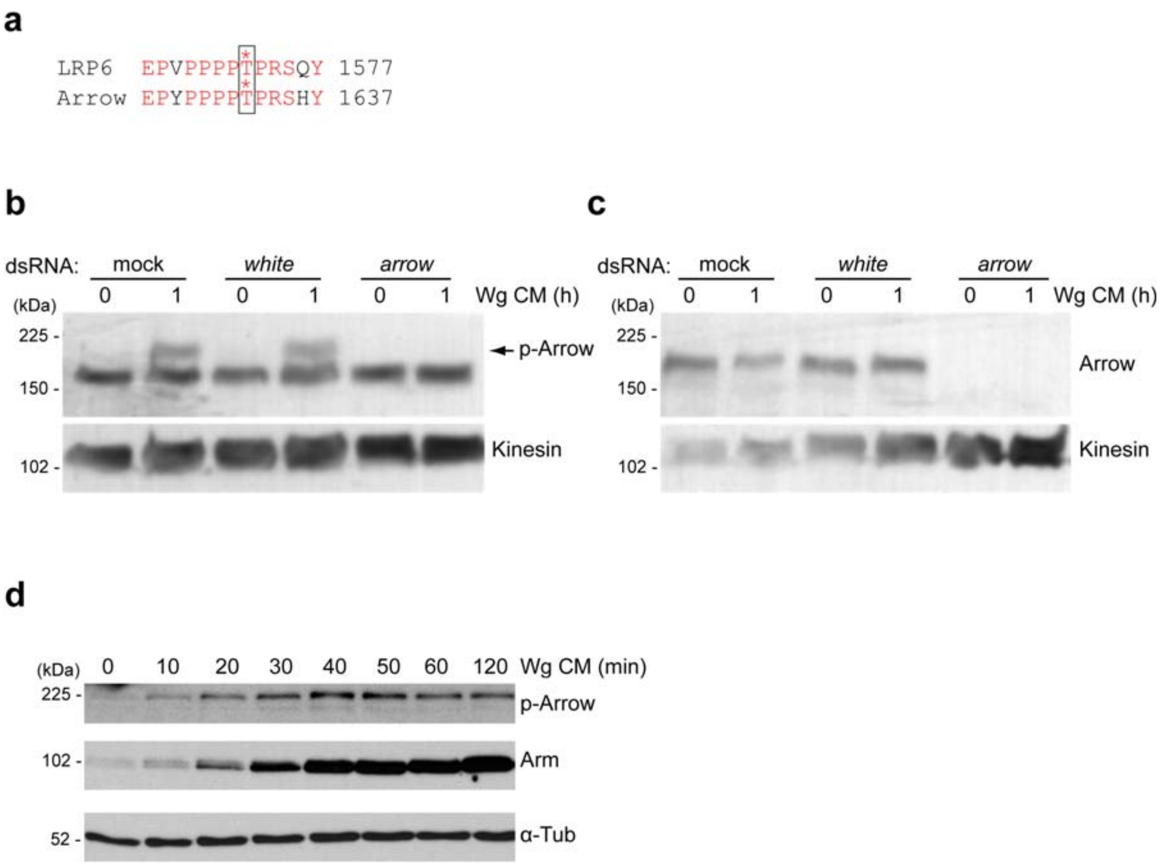

Supplementary Figure 8. **Establishment of a Drosophila cell culture model for Axin regulation following Wingless stimulation.** (a) Alignment showing amino acid conservation in the phosphorylated peptide used to generate the phospho-LRP6 (T1572) antibody with the corresponding region in Drosophila Arrow. Conserved amino acids are shown in red and the boxed asterisk denotes the phosphorylated threonine 1572 residue. (b and c) Specificity of both the phospho-Arrow/LRP6 and Arrow antibodies. S2R+ cells, either mock-treated, or treated with dsRNAs against *white* (negative control) or *arrow (arrow 1 + arrow 2).* After dsRNA treatment, cells were treated with Wg conditioned medium (CM) or control (complete Scheneider's medium) for 1 hour.

Lysates were analyzed by immunoblot using the indicated antibodies. (b) The phospho-Arrow/LRP6 antibody detects two bands in S2R+ lysates, a specific upper band and a non-specific lower band. The specific upper band increases in intensity upon treatment with Wg CM, and is lost upon treatment with *arrow* dsRNAs. The non-specific lower band does not change in intensity in the presence of Wg CM or upon treatment with *arrow* dsRNA. (c) *arrow* dsRNAs result in loss of signal in the immunoblot with Arrow antibody. (d) Treatment of S2R+ cells with Wg CM resulted in increased levels of phospho-Arrow and Armadillo/β-catenin (Arm). Immunoblot analysis of lysates from S2R+ cells treated with Wg CM for the indicated times. Phospho-Arrow levels increase by 10 minutes after Wg stimulation and peak at 40 to 50 minutes. Arm accumulates by 20 minutes after Wg stimulation, and remains elevated for 2 hours. α-Tub or Kinesin were used as loading control.

Fig. S9.

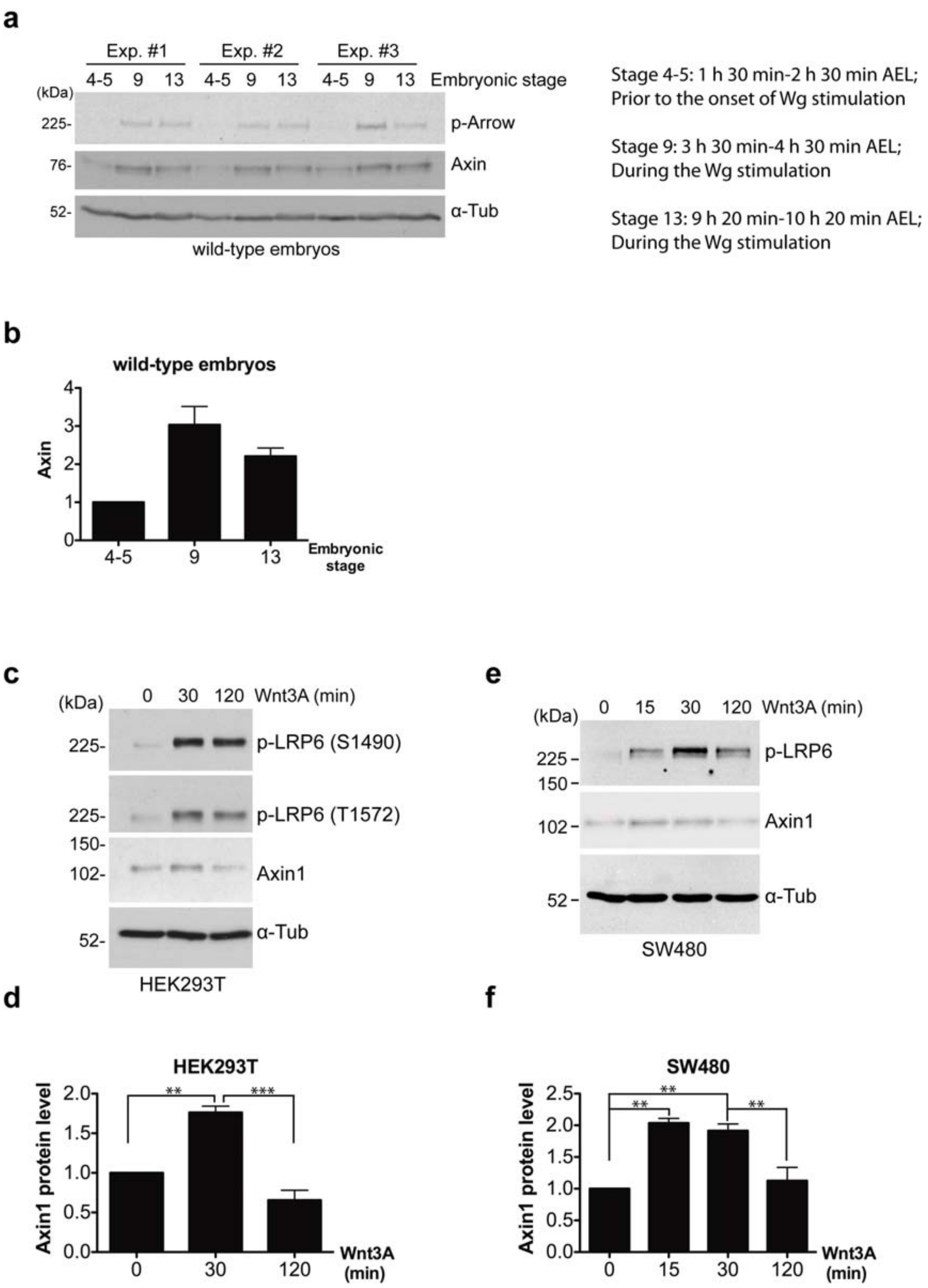
a
Exp. #1
Exp. #2
Exp. #3
4-5 9 13 4-5 9 13 4-5 9 13 Embryonic stage
(kDa)
225-
p-Arrow
76-
Axin
52-
α-Tub
wild-type embryos
Stage 4-5: 1 h 30 min-2 h 30 min AEL;
Prior to the onset of Wg stimulation
Stage 9: 3 h 30 min-4 h 30 min AEL;
During the Wg stimulation
Stage 13: 9 h 20 min-10 h 20 min AEL;
During the Wg stimulation
b
wild-type embryos
Axin
0 1 2 3 4
4-5 9 13
Embryonic stage
c
(kDa)
0 30 120 Wnt3A (min)
225-
p-LRP6 (S1490)
225-
p-LRP6 (T1572)
150-
102-
Axin1
52-
α-Tub
HEK293T
e
(kDa)
0 15 30 120 Wnt3A (min)
225
150
p-LRP6
102
Axin1
52
α-Tub
SW480
d
HEK293T
Axin1 protein level
0.0 0.5 1.0 1.5 2.0
**
***
0 30 120
Wnt3A (min)
f
SW480
Axin1 protein level
0.0 0.5 1.0 1.5 2.0 2.5
**
**
**
0 15 30 120
Wnt3A (min)

Supplementary Figure 9. **Axin is rapidly stabilized and subsequently destabilized following Wnt exposure.** (a and b) Time course of endogenous Axin regulation in response to Wg stimulation. Immunoblot analysis of lysates from wild-type embryos. By comparison with stages 4-5 embryos (prior to the onset of Wg stimulation), the Axin levels in stage 9 embryos (the early phase following Wg exposure) increase about three-fold. Subsequently, the Axin levels decrease in stage 13 embryos (the delayed phase following Wg exposure). Phospho-Arrow levels increase following Wg stimulation. (b) Quantification of relative Axin protein levels from immunoblot analysis in (a). Error bars represent s.e.m. P=0.0459 (one-way ANOVA with Kruskal-Wallis test). (c and e) Time course of Axin regulation in response to Wnt stimulation in human cells. Immunoblot analysis of lysates from HEK293T and SW480 cells treated with human Wnt3A for the indicated times. Phospho-LRP6 (S1490 and T1572) levels increase by 15 to 30 minutes after Wnt3A stimulation. Axin1 levels increase by 15 to 30 minutes and subsequently decrease by 2 hours after Wnt3A stimulation. α-Tub was used as a loading control. (d and f) Quantification of relative Axin1 protein levels from experiments shown in (c and e respectively). Axin1 protein levels increase by approximately two-fold in both cell lines after Wnt3A stimulation. Error bars represent s.e.m. of three independent experiments. (d) **P>0.005, ***P>0.0005 (one-way ANOVA with Tukey's multiple comparison test) (f) **P>0.005 (one-way ANOVA with Tukey's multiple comparison test).

Fig. S10.

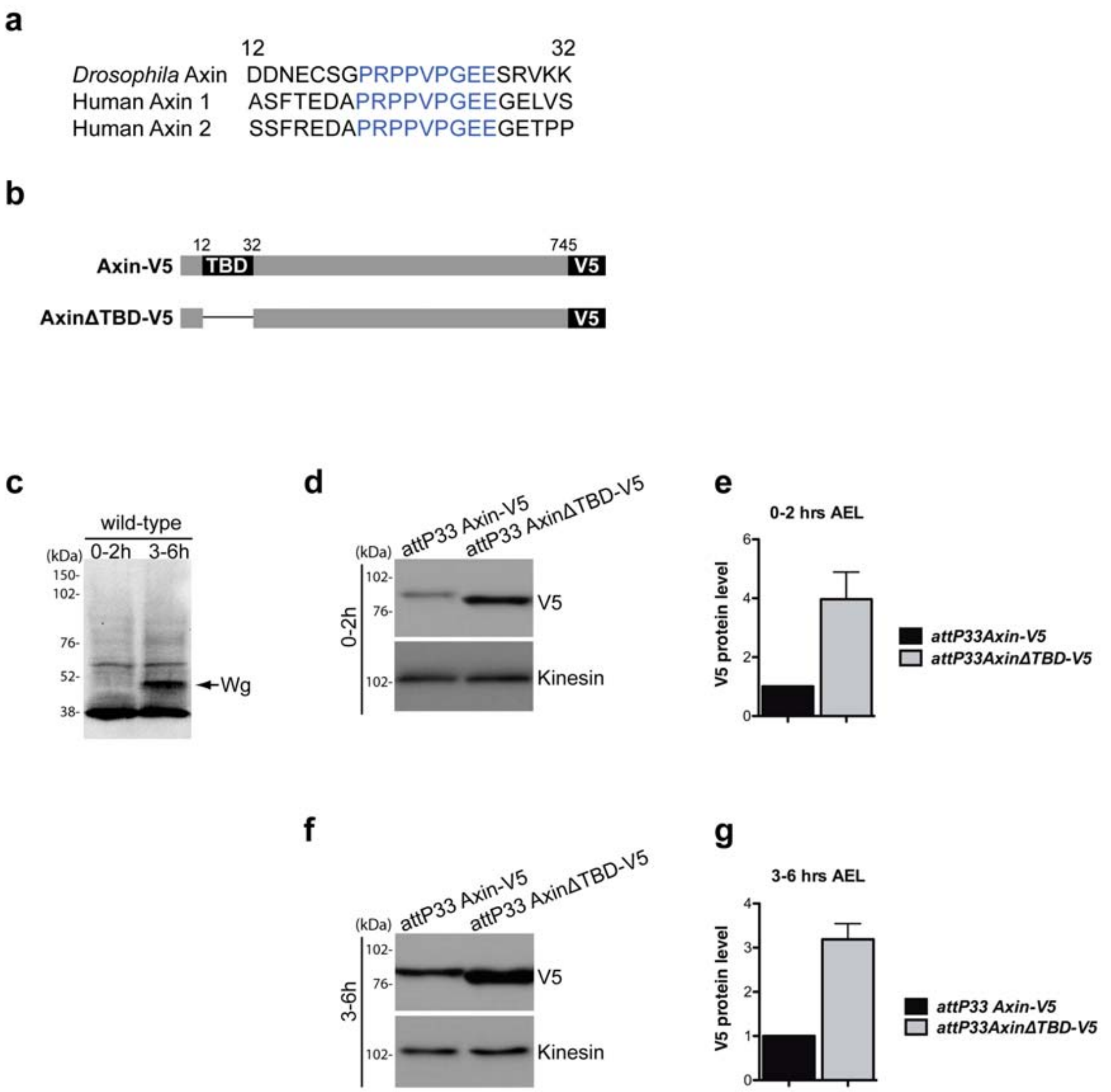

Supplementary Figure 10. The Tankyrase binding domain in Drosophila Axin is required to control basal Axin levels. (a) Alignment showing the amino acid conservation (blue, identical residues) within the Tnks binding domain (TBD) between Drosophila Axin and the two human Axin proteins (Axin1 and Axin2). (b) Schematic representation of Axin-V5 and AxinΔTBD-V5 generated for this study. AxinΔTBD-V5 is a

deletion of 21 residues comprising the TBD of Axin. (c) Immunoblot analysis of lysates from wild-type embryos with Wg antibody. Wg protein is not present in lysates from embryos collected at 0 to 2 hours of development, but is detectable in lysates from embryos collected at 3 to 6 hours of development. (d-g) The TBD in Axin is required for regulation of Axin levels. Immunoblot of lysates from embryos expressing *attP33 Axin-V5 or attP33 AxinΔTBD-V5* driven by the *mat-Gal4* driver. Lysates were prepared from embryos collected at 0-2 hours (d) or 3-6 hours (f) of development, before or after the onset of Wg expression, respectively. Levels of V5 from *attP33 AxinΔTBD-V5* are higher than *attP33 Axin-V5* when expressed under the same conditions at both 0-2 and 3-6 hours of development. Kinesin was used as a loading control. (e, g) Quantification of relative indicated protein levels from experiments shown in (d, f) respectively. Error bars represent s.e.m. of three independent experiments. (e) *P*=0.0241 (Student's *t*-test). (g) *P*=0.0129 (Student's *t*-test).

Fig. S11.

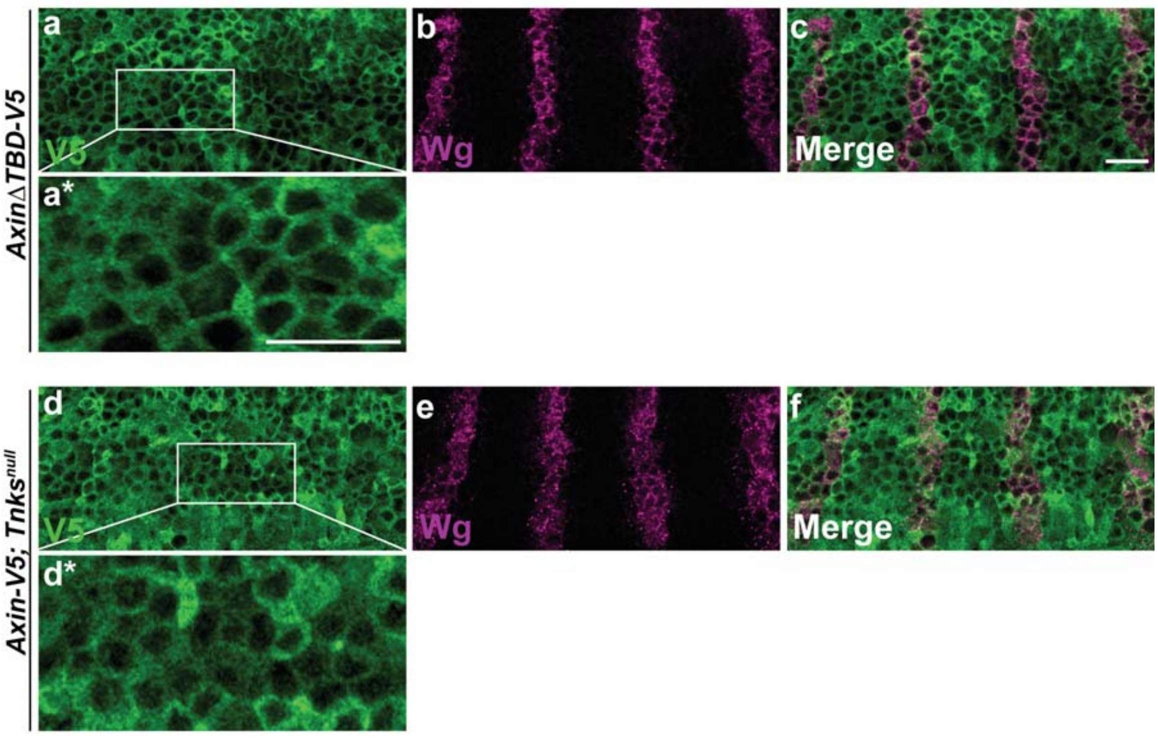

Supplementary Figure 11. Tnks and the Tnks binding site in Axin are required for the initial stabilization of Axin induced by Wingless exposure. (a-c) Confocal images of stage 9 embryos expressing *AxinΔTBD-V5* or *Tnks* null mutant embryos expressing *Axin-V5* driven by *mat-Gal4* driver, stained with V5 and Wg antibodies. V5 staining is uniformly high throughout the embryonic ectoderm; no segmental stripes are present. (a* and d*) Higher magnification of corresponding boxed region. Scale bars represent 25 μm.

Fig. S12.

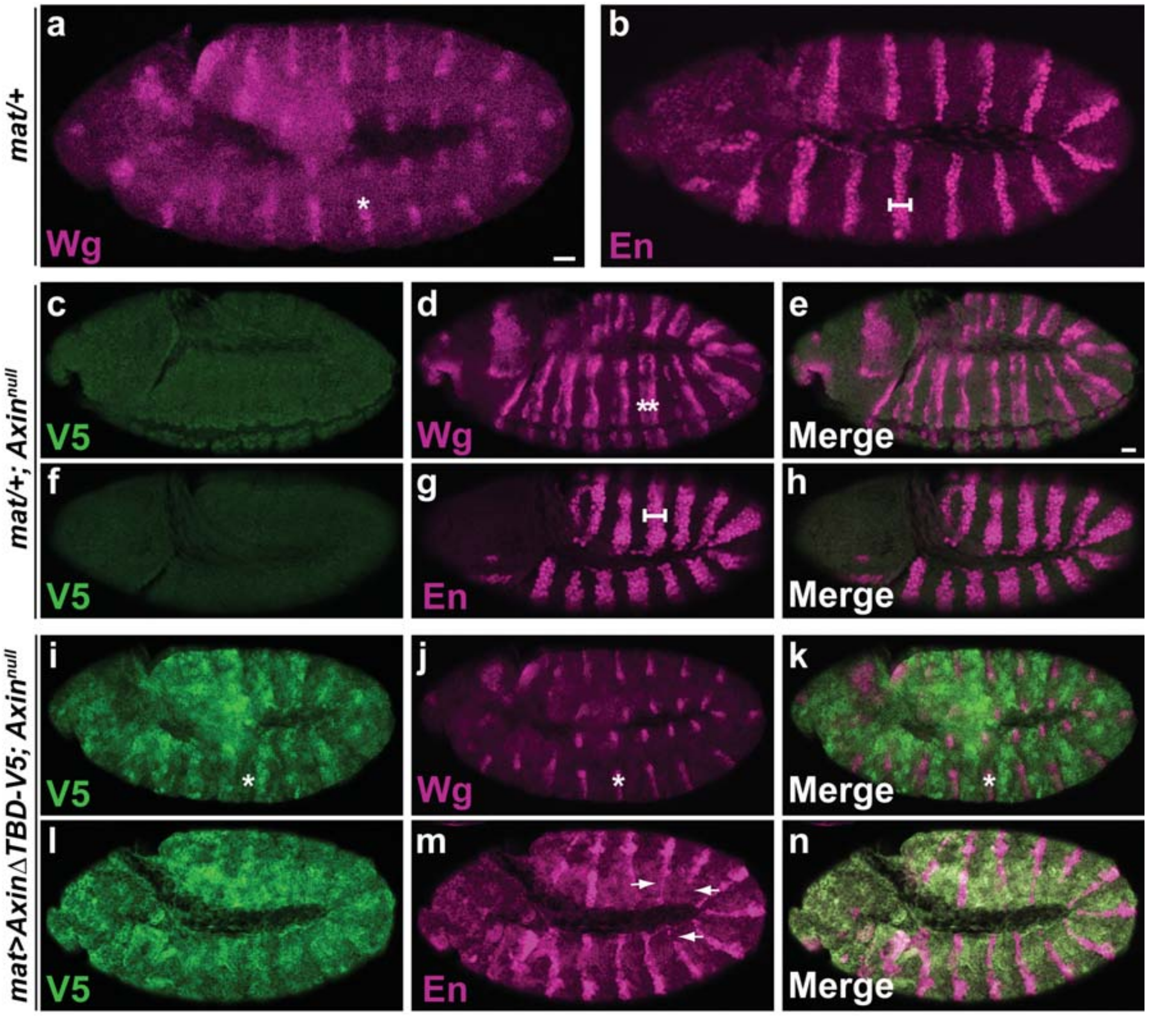

Supplementary Figure 12. The Tnks binding domain promotes the activation of signaling following Wingless exposure. Confocal images of stage 10 embryos stained with V5, En and Wg antibodies. (a and b) Control embryos, displaying the normal number of Wg stripes (a, asterisk) and the normal width of En stripes (b, white bar) within each parasegment. (c-h) In maternal/zygotic *Axin*^{s044230} null mutant embryos, ectopic Wg signaling results in a doubling of the Wg stripes (d, asterisks) and expansion in the width of the En stripes (g, white bar). (i-k) Maternal/zygotic *Axin*^{s044230} null mutant

embryos at stage 10 expressing *AxinΔTBD-V5* driven by the *mat-Gal4* driver. Stripes of AxinΔTBD-V5 are juxtaposed with Wg expressing stripes (asterisk) such that there is no overlap. The normal number of Wg stripes is present, indicating that ectopic Wg signaling has been prevented by expression of *AxinΔTBD-V5*. (l-n) However, staining with En antibody shows that En stripes are reduced in width (arrows). Thus *AxinΔTBD-V5* not only prevents ectopic Wg signaling, but also disrupts endogenous signaling.

Fig. S13.

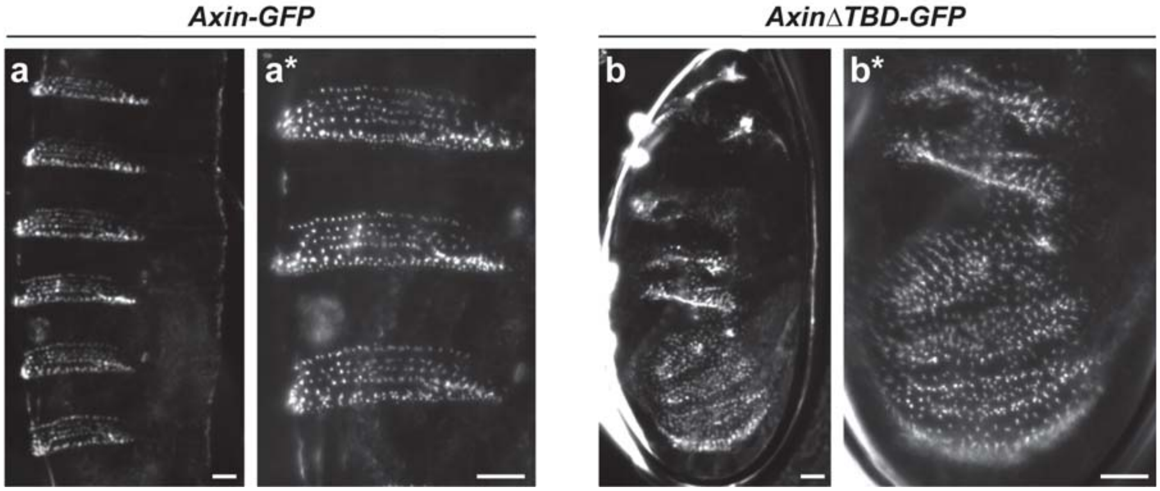

Supplementary Figure 13. The Tnks binding domain in Axin promotes activation of Wingless signaling. Cuticles from embryos expressing either *Axin-GFP* or *AxinΔTBD-GFP* driven by *mat-Gal4*. (a) Larval cuticles reveal normal Wg dependent cell fate specification in embryos expressing *Axin-GFP,* indicated by the presence of naked cuticle that alternates with denticle belts. (b) The Tnks binding domain in Axin promotes Wingless pathway activation. Wingless-dependent cell fates are aberrantly specified in 45% (n=40) of embryos expressing *AxinΔTBD-GFP,* as indicated by a "lawn of denticles" phenotype.

Supplementary Figure 14.

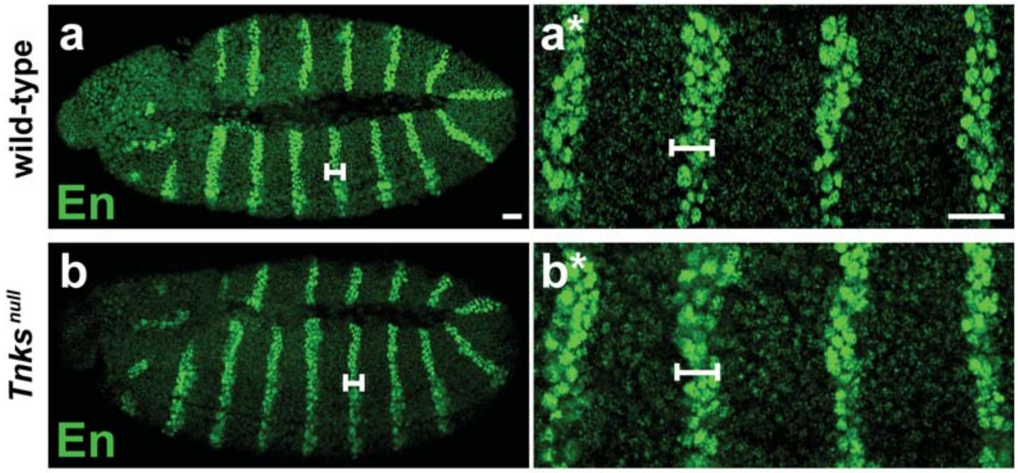

Supplementary Figure 14. In wild-type embryos, *Tnks* loss does not disrupt Wingless signaling. Confocal images of stage 10 embryos stained with En antibody. Genotypes left. In all images, anterior left, dorsal up. (a) In wild-type and (b) *Tnks* null mutant embryos, En is present in stripes that are 2 to 3 cells in width. White bars indicate the width a single En stripe. 96.4 % of wild-type embryos have normal En expression (n=112). 94.7% of *Tnks* mutant embryos have normal En expression (n=57). Images in (a* and b*) are higher magnification views of embryos in (a and b) respectively. Scale bars represent 25 μm. These experiments were performed using two different *Tnks* null alleles, $Tnks^{503}$ and $Tnks^{19}$.

Supplementary Figure 15.

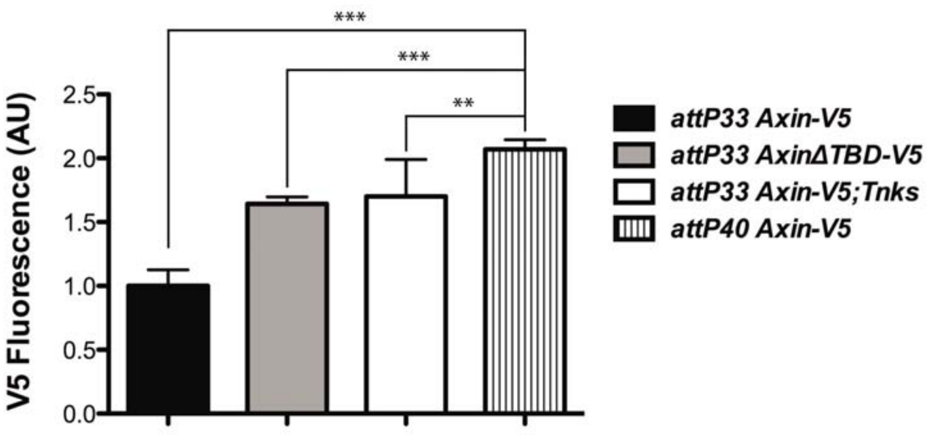

Supplementary Figure 15. Axin levels are regulated by Tnks and the Tnks binding domain in Axin during embryogenesis. Quantification of Axin-V5 fluorescence intensity in cells responding to Wg stimulation in stage 9 embryos co-stained with V5 and Wg antibodies. Comparison of wild-type embryos expressing *attP40 Axin-V5*, *attP33 Axin-V5, attP33 AxinΔTBD-V5,* or *Tnks* null mutant embryos expressing *attP33 Axin-V5,* as indicated on the right. Five embryo samples per each genotype were randomly captured from over hundred embryos. Fluorescence microscope settings remained unchanged for the entire experiment. Bars represent the average intensity of V5 fluorescence (AU) in cells surrounding the Wg stripe in the third segment with respect to the anterior-posterior axis. 50 to 100 measurements were made along the length of each line and averaged. Error bars indicate the s.d. **P>0.005, ***P>0.0005 (one-way ANOVA with Dunnett's multiple comparison test). In the absence of Tnks, or upon deletion of the TBD in Axin, attP33 Axin-V5 levels were increased, but remained lower than attP40 Axin-V5 levels.

Supplementary Figure 16.

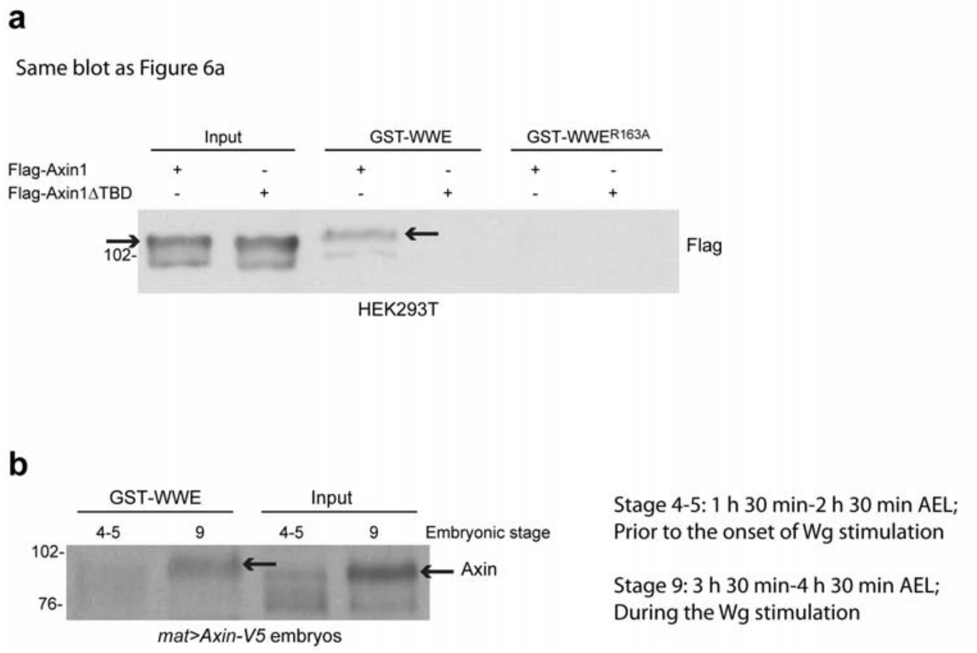

Supplementary Figure 16. ADP-ribosylated Axin pulled down by GST-WWE has altered mobility. (a) Flag-Axin1 is pulled down by GST-WWE has a mobility shift compared with the input in HEK293T cells. Same blot as Figure 6a. See the Figure 6a legend. (b) The ADP-ribosylated Axin is present at increased levels and has a mobility shift relative to the input following Wg stimulation in Drosophila embryos. Embryonic stages 4-5 or 9 expressing *Axin-V5* driven by the *mat-Gal4* driver. Embryonic lysates were pulled down by GST-WWE followed by immunoblot analysis with Axin antibody. Lysates were size-fractionated on 4-15% gradient gel.

Supplementary Figure 17.

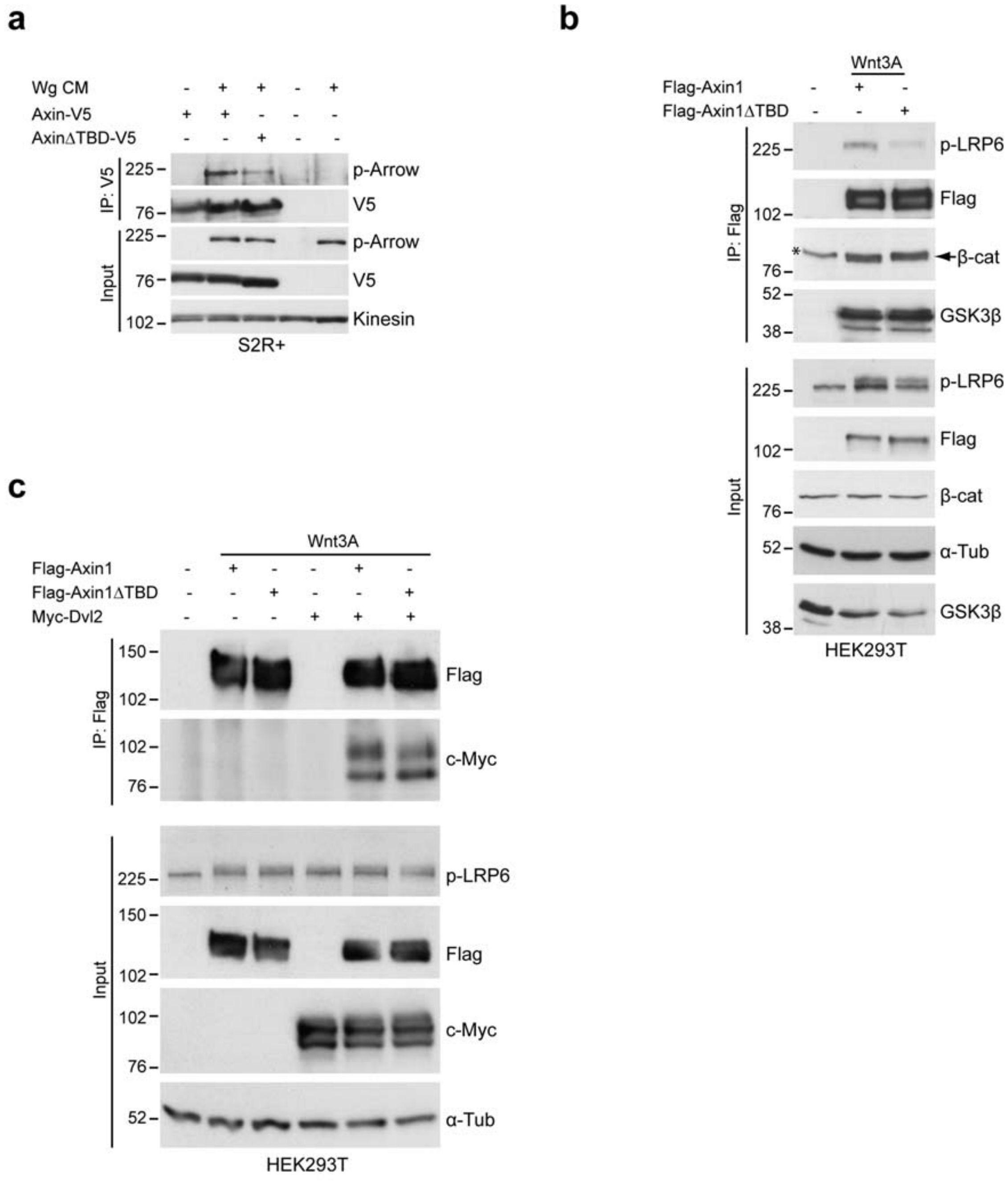

Supplementary Figure 17. ADP-ribosylation enhances the interaction of Axin with phospho-LRP6, but not with β-cat, GSK3β or Dishevelled. (a) The TBD in Drosophila

Axin enhances its Wg-dependent interaction with phospho-Arrow. S2R+ cells were transfected with either Axin-V5 or AxinΔTBD-V5, and were treated subsequently with Wg CM for 1 hour. Lysates were immunoprecipated with V5 antibody, followed by immunoblot analysis with the indicated antibodies. Deletion of the Tnks binding domain of Axin diminishes the interaction between Axin and phospho-Arrow. (b) The TBD in mouse Axin1 enhances its Wnt-dependent interaction with phospho-LRP6, but not with β-catenin or GSK3β. HEK293T cells transfected with either Flag-Axin1 or Flag-Axin1ΔTBD, and subsequently treated with Wnt3A for 30 minutes. Lysates were immunoprecipitated with Flag antibody, followed by immunoblot analysis with indicated antibodies. Both Axin1 and Axin1ΔTBD interact with β-cat or GSK3β. The same blots were analyzed in experiment shown in Fig. 7d. Asterisk indicates non-specific band. (c) The TBD in mouse Axin1 is dispensable for the interaction of Axin with Dishevelled (Dvl). HEK293T cells transfected with Flag-Axin1, Flag-Axin1ΔTBD or Myc-Dvl2, and subsequently treated with Wnt3A for 30 minutes. Lysates were immunoprecipitated with Flag antibody, followed by immunoblot analysis with indicated antibodies. Both Axin1 and Axin1ΔTBD interact with Dvl2. Note: as reported previously, detection of the interaction between Dvl and Axin has often required overexpression of both proteins [2-7]. As we were unable to detect the Axin-Dvl interaction with endogenous proteins, we cannot exclude the possibility that the Axin-Dvl interaction is also regulated by ADP-ribosylation.

Supplementary Figure 18.

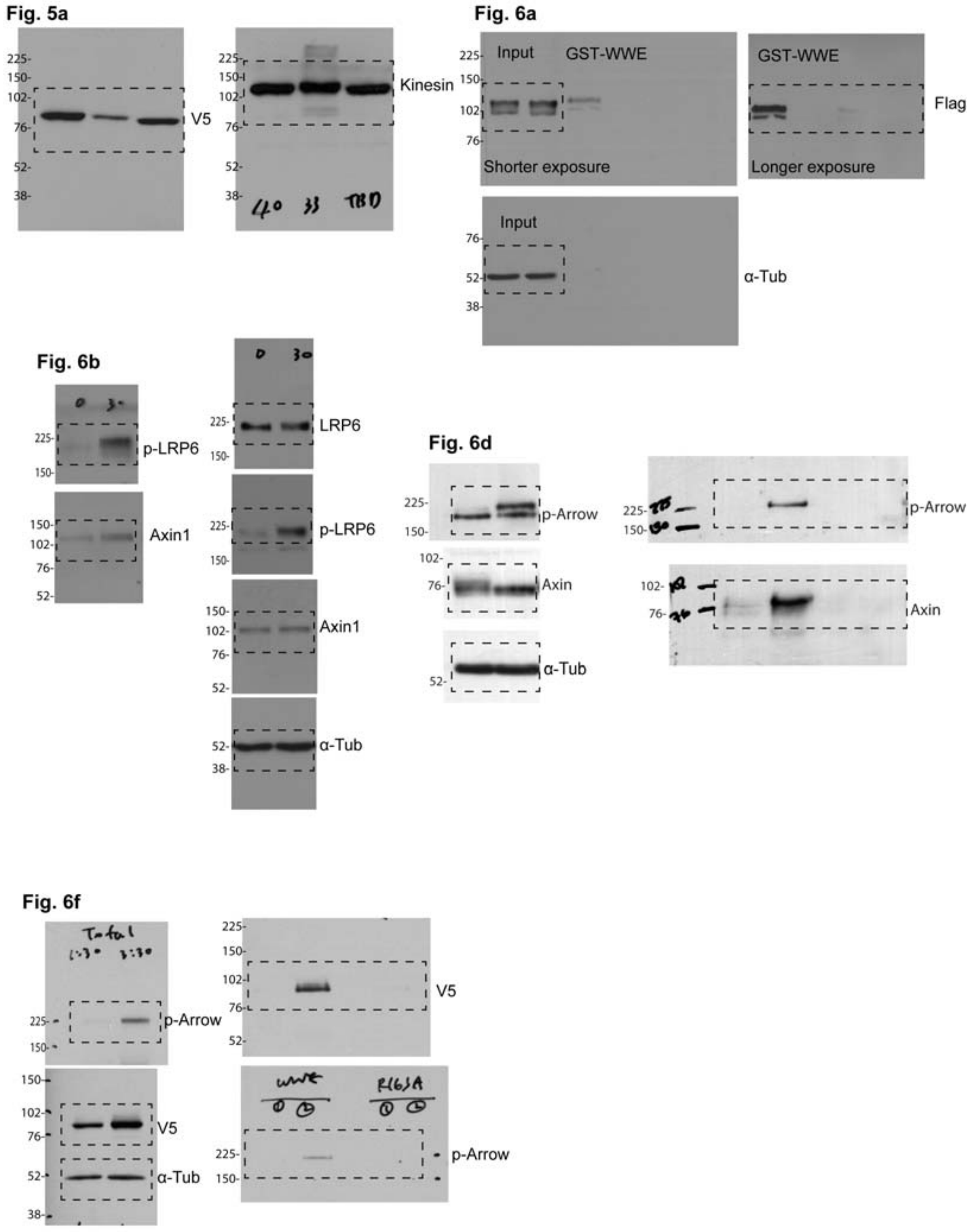

Supplementary Figure 18-2.

Fig. 7a

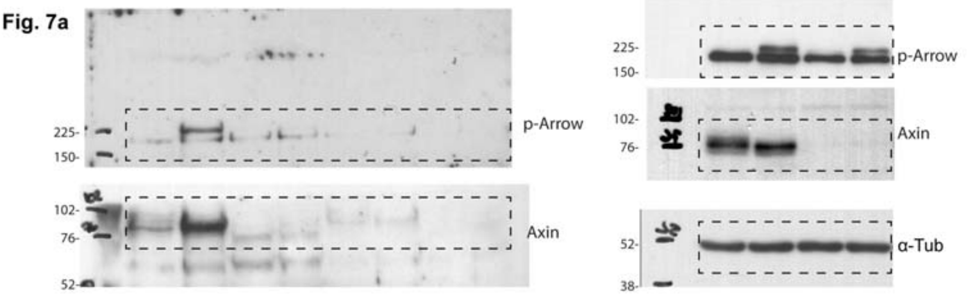

Fig. 7b

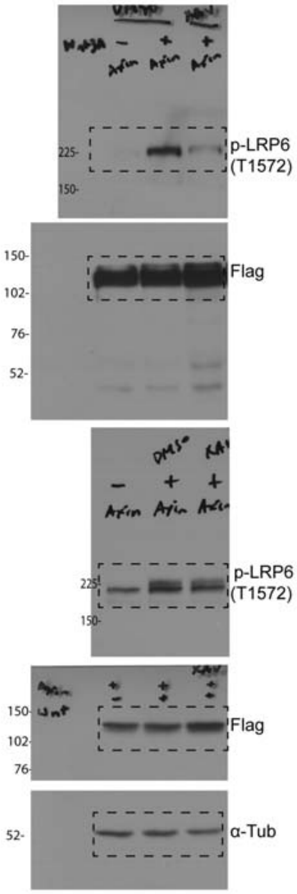

Fig. 7c

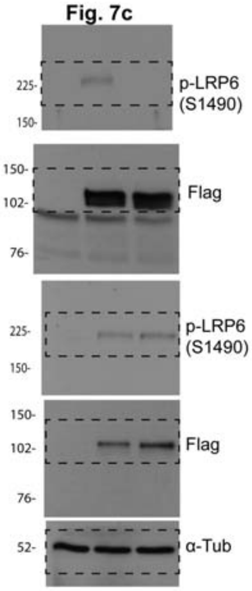

Fig. 7d

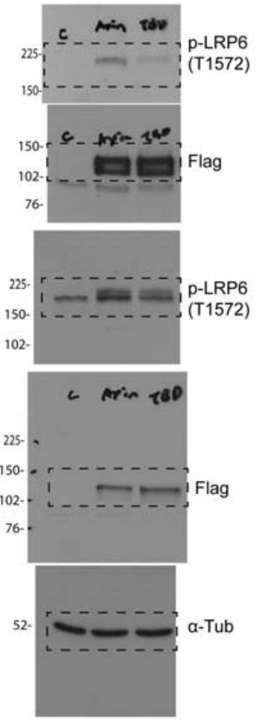

Supplementary Figure 18. Uncropped images of immunoblots.

Supplementary Methods

Drosophila stocks and genetics

The maternal *α4-Gal4:VP16 (mat-Gal4;* line 67; 15*)* driver contains the maternal *tubulin* promoter from *αTub67C* and the 3' UTR from *αTub84B* [8, 9]. Other stocks used were: $Tnks^{19}$, $Tnks^{503}$ [10], *FRT82B* ovo^{D1} [11] (Bloomington Drosophila Stock Center (BDSC)), *hsFLP1* [12] (BDSC), *arm-Gal4* [13], and wg^{CX4} [14].

Axin mutant germline clone embryos expressing *Axin-V5* driven by *mat-Gal4* were generated using the *FLP-DFS* technique [11]. First and second instar larvae of the genotypes listed below were heat shocked at 37°C during first or second instar for 1 to 2 hours, and then as adults were crossed to *UAS-Axin-V5;* $Axin^{s044230}$*/TM6B* males. The presence of the *y*+ marker at the *attP* integration site [15] facilitated identification of females carrying the *UAS-Axin-V5* transgene.

y w hsFLP1 / +; mat-Gal4 / UAS-Axin-V5; FRT82B ovo^{D1} */ FRT82B* $Axin^{s044230}$

y w hsFLP1 / +; mat-Gal4 / UAS-AxinΔTBD-V5; FRT82B ovo^{D1}*/ FRT82B* $Axin^{s044230}$

Embryonic hatch rate and cuticle preparation

Embryos were collected for 1 hour and incubated at 25°C for an additional 24 hours before the hatch rate was scored. At least 200 embryos of each genotype were analyzed. Larval cuticles were prepared as described [1].

Immunostaining and immunoblotting

For immunostaining, embryos were fixed in 3.7% formaldehyde [16] and rehydrated in PBT (phosphate buffered saline [PBS], 0.1% Tween-20, and 1% bovine serum albumin [BSA]). Following incubation for one hour in blocking solution (PBS, 0.1% Tween-20, 10% BSA), embryos were incubated overnight at 4°C with primary antibodies in PBT. After washing with PTw (PBS, 0.1% Tween-20), embryos were incubated with secondary antibodies for one hour at room temperature. Embryos were then washed with PTw and mounted in Prolong Gold (Invitrogen). Fluorescent images were obtained on a Nikon NIS confocal microscope and an Axioskop 2 plus fluorescence microscope, and processed and assembled using Adobe photoshop CS5 and Adobe illustrator CS5.

For immunoblotting, embryos or cultured mammalian cells were lysed in lysis buffer (50 mM Tris-HCl [pH 8.0], 100 mM NaCl, 1% NP-40, 10% glycerol, 1.5 mM EDTA [pH 8.0]), supplemented with phosphatase and protease inhibitor cocktail (1:100, Thermo Scientific) and 1 μM of the poly(ADP-ribose) glycohydrolase inhibitor ADP-HPD (Enzo Life Sciences). Lysates were size-fractionated on 6, 8 or 4-15% SDS-PAGE and transferred to PVDF membranes that were blocked in TBS with 5% skim milk or 5% BSA. Membranes were incubated overnight at 4°C or one hour at room temperature with primary antibodies. After washing with TBST (TBS, 0.1% Tween-20) or PBST (PBS, 0.1% Tween-20), membranes were incubated with the appropriate HRP-conjugated secondary antibodies for 1 hour at room temperature. Membranes were then washed and developed using ECL reagent (250 mM luminol, 90 mM p-coumaric acid, 100 mM Tris-HCL [pH 8.5], 30% hydrogen peroxide) or West Femto substrate (Thermo Scientific). Protein levels were quantified and normalized using Image J software (National Institutes of Health). *Drosophila* cultured cells were lysed in 4X sample buffer supplemented with 1 M DTT. Lysates were normalized by volume and protein

concentration, subsequently resolved by SDS–PAGE, transferred onto PVDF membranes and probed with the indicated antibodies. Uncropped western blots are shown in Supplementary Fig. 18.

Immunoprecipitation

For immunoprecipitation experiments with lysates from mammalian cell lines, lysates were prepared as described above and incubated with the mouse anti-Flag affinity gel (Sigma) for 3 hours at 4°C or mouse anti-Flag (Sigma) overnight at 4°C followed by addition of Protein G Plus-Agarose beads (Santa Cruz Biotechnology) for 1 hour at 4°C. Beads were washed four times with lysis buffer and boiled with 2X SDS sample buffer (4% SDS, 20% glycerol, 120 mM Tris-HCl [pH 6.8], 0.02% bromophenol blue, 10% DTT), resolved by SDS-PAGE, and immunoblotted with the indicated antibodies. For immunoprecipitation experiments with lysates from the S2R+ cell line, cells were harvested 48 hours after transfection, washed with 1X PBS, then lysed in lysis buffer (as above) supplemented with 1 μM APD-HPD (Enzo Life Sciences) and phosphatase and protease inhibitor cocktail (1:100, Thermo Scientific). Lysates were incubated with mouse anti-V5 antibody (Invitrogen) overnight at 4°C, followed by addition of protein A/G-sepharose beads (Santa Cruz Biotechnology) for 1 hour at 4°C. Beads were washed three times with wash buffer (50 mM Tris-HCl [pH 8.0], 150 mM NaCl, 1% NP-40, 10% Glycerol, 1.5 mM EDTA [pH 8.0]) supplemented with 1 μM APD-HPD and phosphatase and protease inhibitor cocktail (1:100), and boiled with 4X sample buffer supplemented with 1M DTT. Samples were resolved by SDS-PAGE and immunoblotted with the indicated antibodies.

Quantification of fluorescence intensity

Axin-V5 fluorescence levels in stage 9 embryos co-stained with V5 and Wg antibodies were quantified using NIS-Elements software (Nikon). A user-drawn single line at the position of the Wg stripe in the third parasegment with respect to the anterior-posterior axis was used to calculate the mean fluorescence intensity of the V5 signal. 50 to 100 measurements were obtained along the length of each line and averaged.

Cell culture, transfection, and treatment with reagents

HEK293T and SW480 cells were cultured in Dulbecco's modified Eagle's medium (Corning Cellgro) supplemented with 10% fetal bovine serum (FBS, Gibco) and 0.1 mg/mL penicillin/streptomycin (Invitrogen) at 37°C under humidified air containing 5% CO_2. Cells were transfected using Lipofectamine 2000 (Invitrogen), according to the manufacture's instructions, and harvested 24 or 48 hours after transfection.

S2R+ cells and S2-Tub-wg cells (Drosophila Genomics Resource Center) were maintained at 25°C in Schneider's complete medium: Schneider's *Drosophila* medium with L-glutamine (Gibco) supplemented with 10% FBS (Gibco) and 0.1 mg/mL penicillin/streptomycin (Invitrogen). Cells were transiently transfected using calcium-phosphate DNA precipitation [17].

For generating Wg conditioned medium (Wg CM), S2-Tub-wg cells were grown to confluence in Scheneider's complete medium, then split 1:3 and incubated at 25°C for 72 hours. Cells were then re-suspended in the Scheneider's complete medium in which they had been cultured, and centrifuged at 1000 x rpm for 5 minutes at room temperature; the supernatant was centrifuged again at 5000 x rpm for 5 minutes at room temperature. The resulting supernatant was stored at 4°C. For treatment with Wg CM, cells were washed 1X with serum-free, antibiotic-free, Schneider's medium. Wg CM or

the complete medium control was added, and cells were incubated at 25°C for the specified times.

Human cells were treated with 5 μM XAV939 (Cayman Chemical) for 24 hours and/or 100 ng/ml Wnt3A (human recombinant, StemRD) for the specified times.

Plasmids

Plasmids used for transfection of human cell lines were *pCS2+ Flag-mouse Axin1* [18] and *pCS2+ Flag-mouse Axin1ΔTBD*. To generate the *pCS2+ Flag-mouse Axin1ΔTBD* plasmid, residues P-26 through E-34 were deleted by PCR-based mutagenesis of *pCS2+ Flag-mouse Axin1* using the oligonucleotide: 5'-GATGCCGGAGAACTGGTATCTACTGAT-3'. The resulting Flag-mouse *AxinΔTBD* fragment was digested with *ClaI* and *BglII,* and then inserted into the *pCS2+ vector* at the *ClaI* and *BglII* sites.

Plasmids used for transfection of *Drosophila* cells were *pAc5.1-Axin-V5* and *pAc5.1-AxinΔTBD-V5*. To generate the *pAc5.1-Axin-V5* and *pAc5.1-AxinΔTBD-V5* plasmids: fragments encoding *Axin-V5* and *AxinΔTBD-V5* from *pUASTattB-Axin-V5* and *pUASTattB-AxinΔTBD-V5* respectively, were digested using *KpnI* and *XbaI*. The resulting fragments were inserted into the *pAc5.1* vector (Invitrogen) at the *KpnI* and *XbaI* sites.

dsRNA generation and RNAi-mediated knockdown

The generation of double-stranded RNAs (dsRNAs) and dsRNA-mediated knockdown was performed as described previously [19]. Briefly, DNA templates of 200-900 nucleotides in length targeting *Axin*, *arrow*, or *white* (negative control) were generated by

PCR from genomic DNA extracted from S2R+ cells. PCR templates contained T7 promoter sequences on both ends. The DNA templates were amplified using the following primer pairs: *Axin 29423* [forward 5'-T7-TCTTAAAGAGCTCGACCCCA-3' and reverse 5'-T7-TAATTGCCTTCCAAATTCGC-3' (sequence from *Drosophila* RNAi Screening Center)]; *Axin 14120* [forward 5'-T7-TCGGATTTCCAGTCTTCTTTT-3' and reverse 5'-T7-CTCTACATCCAGCAGATGTC-3' (sequence from *Drosophila* RNAi Screening Center)]; *arrow #1* [forward 5'-T7-CGATATGGATGAGCCGTATGCGGT-3' and reverse 5'-T7-CCGTTGATGTCAATGGCATCGA-3']; *arrow #2* [forward 5'-T7-GAGAACTGCGCCGATGGAGCT-3' and reverse 5'-T7-GCAGGAGGATTCCGGCACAATG-3']; *white* forward [5'-T7-ACCTGTGGACGCCAAGG-3' and reverse 5'-T7- AAAAGAAGTCGACGGCTTC-3' (sequence from [20]]. dsRNAs were transcribed from PCR generated templates using the T7 Megascript kit (Ambion) according to manufacturer's instructions. For RNAi-mediated knockdown, S2R+ cells were plated in 10 cm plates with 2.5 mL of serum-free, antibiotic-free Schneider's medium + L-glutamine. 25 ug of each dsRNA was added to the medium and cells were incubated with gentle rotation at room temperature for 1 hour. Following incubation, 2.5 mL of complete medium were added and cells were incubated at 25°C. After 24 hours, medium was removed from the cells. This procedure was repeated once every 24 hours for a total of 96 hours.

Supplementary Reference

1. Wieschaus, E. & Nüsslein-Volhard, C. *In Drosophila: A practical approach*. (IRL Press, Oxford; 1986).

2. Kim, W.T. *et al.* Dual functions of DP1 promote biphasic Wnt-on and Wnt-off states during anteroposterior neural patterning. *The EMBO journal* **31**, 3384-3397 (2012).
3. Schwarz-Romond, T., Metcalfe, C. & Bienz, M. Dynamic recruitment of axin by Dishevelled protein assemblies. *Journal of cell science* **120**, 2402-2412 (2007).
4. Fiedler, M., Mendoza-Topaz, C., Rutherford, T.J., Mieszczanek, J. & Bienz, M. Dishevelled interacts with the DIX domain polymerization interface of Axin to interfere with its function in down-regulating beta-catenin. *Proceedings of the National Academy of Sciences of the United States of America* **108**, 1937-1942 (2011).
5. Kishida, S. *et al.* DIX domains of Dvl and axin are necessary for protein interactions and their ability to regulate beta-catenin stability. *Molecular and cellular biology* **19**, 4414-4422 (1999).
6. Peterson-Nedry, W. *et al.* Unexpectedly robust assembly of the Axin destruction complex regulates Wnt/Wg signaling in Drosophila as revealed by analysis in vivo. *Developmental biology* **320**, 226-241 (2008).
7. Smalley, M.J. *et al.* Interaction of axin and Dvl-2 proteins regulates Dvl-2-stimulated TCF-dependent transcription. *The EMBO journal* **18**, 2823-2835 (1999).
8. Benton, R. & St Johnston, D. Drosophila PAR-1 and 14-3-3 inhibit Bazooka/PAR-3 to establish complementary cortical domains in polarized cells. *Cell* **115**, 691-704 (2003).
9. Hacker, U. & Perrimon, N. DRhoGEF2 encodes a member of the Dbl family of oncogenes and controls cell shape changes during gastrulation in Drosophila. *Genes Dev* **12**, 274-284 (1998).
10. Wang, Z. *et al.* The ADP-ribose polymerase Tankyrase regulates adult intestinal stem cell proliferation during homeostasis in Drosophila. *Development* **In press** (2016).
11. Chou, T.B. & Perrimon, N. Use of a yeast site-specific recombinase to produce female germline chimeras in Drosophila. *Genetics* **131**, 643-653 (1992).
12. Golic, K.G. & Lindquist, S. The FLP recombinase of yeast catalyzes site-specific recombination in the Drosophila genome. *Cell* **59**, 499-509 (1989).
13. Sanson, B., White, P. & Vincent, J.P. Uncoupling cadherin-based adhesion from wingless signalling in Drosophila. *Nature* **383**, 627-630 (1996).
14. Baker, N.E. Molecular cloning of sequences from wingless, a segment polarity gene in Drosophila: the spatial distribution of a transcript in embryos. *The EMBO journal* **6**, 1765-1773 (1987).
15. Markstein, M., Pitsouli, C., Villalta, C., Celniker, S.E. & Perrimon, N. Exploiting position effects and the gypsy retrovirus insulator to engineer precisely expressed transgenes. *Nat Genet* **40**, 476-483 (2008).

16. Vorwald-Denholtz, P.P. & De Robertis, E.M. Temporal pattern of the posterior expression of Wingless in Drosophila blastoderm. *Gene Expr Patterns* **11**, 456-463 (2011).
17. Graham, F.L. & van der Eb, A.J. A new technique for the assay of infectivity of human adenovirus 5 DNA. *Virology* **52**, 456-467 (1973).
18. Tamai, K. *et al.* A mechanism for Wnt coreceptor activation. *Molecular cell* **13**, 149-156 (2004).
19. Rogers, S.L. & Rogers, G.C. Culture of Drosophila S2 cells and their use for RNAi-mediated loss-of-function studies and immunofluorescence microscopy. *Nat Protoc* **3**, 606-611 (2008).
20. Zhang, Y. *et al.* RNF146 is a poly(ADP-ribose)-directed E3 ligase that regulates axin degradation and Wnt signalling. *Nat Cell Biol* **13**, 623-629 (2011).
